# A Dual Role for SOX10 in the Maintenance of the Postnatal Melanocyte Lineage and the Differentiation of Melanocyte Stem Cell Progenitors

**DOI:** 10.1371/journal.pgen.1003644

**Published:** 2013-07-25

**Authors:** Melissa L. Harris, Kristina Buac, Olga Shakhova, Ramin M. Hakami, Michael Wegner, Lukas Sommer, William J. Pavan

**Affiliations:** 1 Genetic Disease Research Branch, National Human Genome Institute, National Institutes of Health, Bethesda, Maryland, United States of America; 2 Department of Genetics, University of Georgia, Athens, Georgia, United States of America; 3 Cell and Developmental Biology, Institute of Anatomy, University of Zurich, Zurich, Switzerland; 4 School of Systems Biology, National Center for Biodefense and Infectious Diseases, George Mason University, Manassas, Virginia, United States of America; 5 Institut für Biochemie, Emil-Fischer-Zentrum, Universität Erlangen-Nürnberg, Erlangen, Germany; University of Iceland, Iceland

## Abstract

During embryogenesis, the transcription factor, *Sox10*, drives the survival and differentiation of the melanocyte lineage. However, the role that *Sox10* plays in postnatal melanocytes is not established. We show in vivo that melanocyte stem cells (McSCs) and more differentiated melanocytes express SOX10 but that McSCs remain undifferentiated. *Sox10* knockout (*Sox10^fl^; Tg(Tyr::CreER)*) results in loss of both McSCs and differentiated melanocytes, while overexpression of *Sox10* (*Tg(DctSox10)*) causes premature differentiation and loss of McSCs, leading to hair graying. This suggests that levels of SOX10 are key to normal McSC function and *Sox10* must be downregulated for McSC establishment and maintenance. We examined whether the mechanism of *Tg(DctSox10)* hair graying is through increased expression of *Mitf*, a target of SOX10, by asking if haploinsufficiency for *Mitf* (*Mitf^vga9^*) can rescue hair graying in *Tg(DctSox10)* animals. Surprisingly, *Mitf^vga9^* does not mitigate but exacerbates *Tg(DctSox10)* hair graying suggesting that MITF participates in the negative regulation of *Sox10* in McSCs. These observations demonstrate that while SOX10 is necessary to maintain the postnatal melanocyte lineage it is simultaneously prevented from driving differentiation in the McSCs. This data illustrates how tissue-specific stem cells can arise from lineage-specified precursors through the regulation of the very transcription factors important in defining that lineage.

## Author Summary

The melanocyte stem cells (McSCs) that reside in the hair follicle are critical for generating the melanocytes that will differentiate and produce pigment for the hair during successive rounds of hair growth. The inappropriate maintenance of McSCs results in hair graying. Thus, our understanding of McSC biology is enhanced through the study of hair graying mouse models. We have discovered that sustained expression of the transcription factor, *Sox10*, in the melanocyte lineage results in loss of McSCs and consequently leads to premature hair graying. Through the use of mouse transgenics, we demonstrate that by changing *Sox10* levels, melanocytes of the hair can preserve their ability to survive and produce normally pigmented hairs while also allowing a portion of them to fulfill the role of an undifferentiated McSC. We also discovered that *Mitf*, a downstream target of SOX10 and the master regulatory transcription factor for melanogenesis, appears to participate in McSC maintenance, perhaps by the negative regulation of SOX10-dependent processes. These observations raise the idea that adult stem cells, like McSCs, may rely on cell type specific transcription factors for their specification and survival, but that these transcriptions factors also have to be carefully regulated to maintain a stem cell fate.

## Introduction

In the adult animal, tissue-specific stem cells exist in a number of organs and function to sustain these tissues during normal homeostasis. However, our understanding of the origin and establishment of tissue-specific stem cells during organogenesis is incomplete. Using melanocytes as a model, we investigated the process of lineage-specific stem cell fate acquisition by examining the role of the transcription factor SOX10 in the formation of the melanocyte stem cell (McSC) within the mouse hair follicle.

Melanocytes of the hair follicle have gained increasing attention for studying cell-specific contributions to organ development and maintenance. Individual hair follicles act as ‘mini-organs’ [1], and each contains melanocytes that provide pigment to the hair shaft concomitantly with hair cycling. Two primary subpopulations of follicular melanocytes exist and are defined by their anatomical location—McSCs remain in hair bulge, whereas the terminally-differentiated and pigmented melanocytes reside in the transient hair bulb region [2], [3]. Identification of each of these subpopulations has been defined molecularly, in part through the use of immunohistochemistry [4]–[7]. Disruption of McSC function results in hair graying, a non-lethal and visible phenotype, and gray-haired mouse models have been used successfully to study adult stem cell establishment and maintenance [7]–[11].

The most critical time point for establishing McSCs appears to be during hair morphogenesis. Studies using the KIT-blocking antibody, ACK, to deplete melanocyte populations perinatally show that McSCs inhabit hair follicles around P4, demonstrated by the fact that they survive independent of KIT-signaling and are sufficient to restore coat color pigmentation [2], [6]. Many of the melanogenic genes expressed by melanoblasts or bulb melanocytes exist at low/absent levels in McSCs. This distinction arises between stages 6 and 8 of hair follicle morphogenesis (∼P4–8) and is indicated by the loss of ki67 expression and the downregulation of MITF, TRP1, TYR, and SOX10 within presumptive McSCs [4], [7], [12]. Although McSCs are not responsible for pigmenting the first morphogenetic hair [3], they are retained within the hair bulge while melanocytes of the hair bulb undergo apoptosis during hair regression [13], [14]. These McSCs then function to regenerate bulb melanocytes during subsequent hair cycles [8].

The subpopulation-specific expression of the transcription factor *Sox10*, where it is expressed in melanoblasts of the skin and melanocytes of the hair bulb but absent from McSCs, suggests that transcriptionally downregulating *Sox10* is the mechanism by which melanoblasts acquire a McSC fate. This hypothesis fits well with the known function of *Sox10* as a transcription factor that participates in melanocyte differentiation by upregulating *Mitf*, the master regulatory gene for melanogenesis. The loss of melanin synthesis proteins, TRP1 and TYR, within presumptive McSCs further supports this idea since SOX10 transcriptionally activates these genes, and that TYR is required by mouse melanocytes to generate pigment [15]–[18]. In the mouse, *Sox10* is expressed during neural crest development and its loss embryonically results in several neurocristopathies, including congenital hypopigmentation [19]–[24]. However, perinatal lethality in *Sox10* null mice has precluded functional analysis of *Sox10* in adult melanocytes. Using conditional transgenics we can now explore the role of *Sox10* postnatally in the melanocytes of the mouse hair follicle.

Here we report that postnatal mouse melanocytes both express and require *Sox10* for normal hair pigmentation. However, constitutive expression of *Sox10* by McSCs disrupts their maintenance by driving their premature differentiation. We also demonstrate that *Mitf* contributes to this regulation, likely through a negative feedback mechanism. Together, these data support the theory that transcription factors responsible for the specification of lineage-defined precursors can later participate in the specification and maintenance of stem cells derived from those precursors.

## Results

### SOX10 is retained postnatally by McSCs and differentiated melanocytes

The expression of SOX10 within the postnatal McSCs and differentiated melanocytes of the hair bulb was compared to the expression of the melanocyte marker, dopachrome tautomerase (DCT). In this study, we define the McSC population by several characteristics: cells that exist within the hair bulge, are capable of self-renewal, and can give rise to melanocyte progenitors that colonize the newly developing hair bulb. Previously, we have shown that the transgenic line, *Tyr::CreERT2*, can target cells with these properties when induced either during postnatal development or within adults [25]. To specifically demonstrate that DCT marks this McSC population, we performed a similar lineage mapping analysis here (Fig. S1). *Tyr::CreERT2; Rosa26^tm1sor^* pups were given a pulse of tamoxifen (TAM) on postnatal days 2 and 3 (P2–3), and assessed for recombined cells by β-galactosidase staining. Melanocytes in *Tyr::*CreERT2 mice express CRE at P2, and within the same hair cycle as TAM treatment (P14), we observed LacZ^+^ cells in the hair bulge and bulb (Fig. S1A–B). This suggests by anatomical position that we have targeted both McSCs and differentiated melanocytes. We further confirmed that these LacZ^+^ bulge cells are McSCs by challenging them to repopulate new hairs after hair plucking. Hair plucking eliminates all differentiated melanocytes leaving only the McSCs to replenish newly generated hairs. Indeed, seven days after initiating a new hair cycle (7 days post plucking, 7dpp) we observed the retention of LacZ^+^ cells in the hair bulge and LacZ^+^ progeny in the hair bulb (Fig. S1C). This confirms that these LacZ^+^ bulge cells are indeed McSCs. We further analyzed these LacZ^+^ McSCs with immunolabeling (Fig. S1D) and confirm that nearly all (97%) express DCT. Thus for the remainder of our analysis we refer to DCT^+^ cells within the hair bulge as the McSC population.

**Figure 1:**
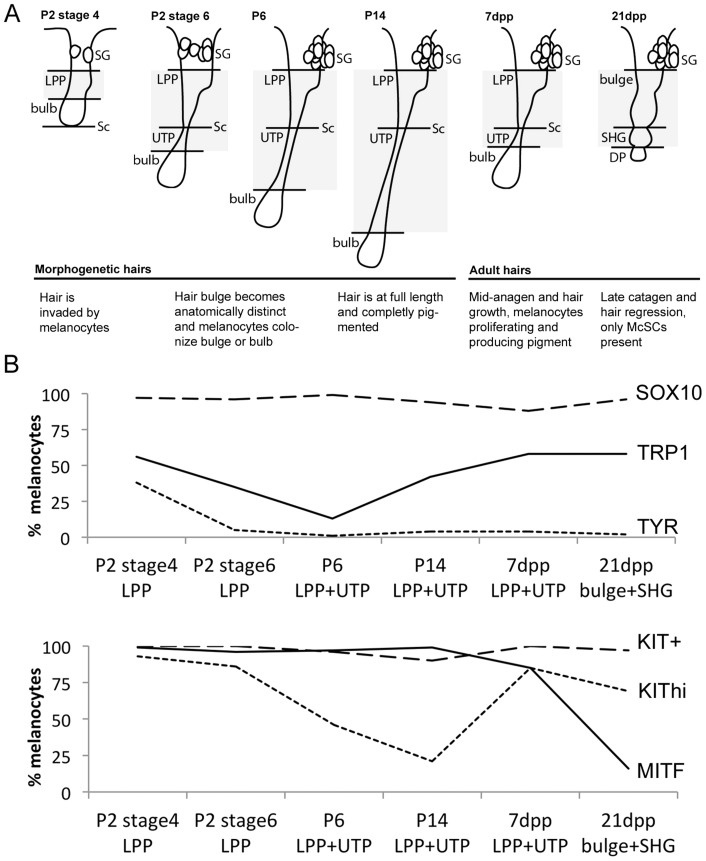
Differentiation status of LPP and UTP melanocytes varies with hair cycling. (A) Schematic of hair follicles during hair morphogenesis (P2 stage 4; P2 stage 6, P6, and P14) and adult hair cycling (7dpp, and 21dpp). Grayed area represents anatomical regions quantified in B. (B) Percent of melanocytes that double-label with DCT and the indicated marker. Counts include melanocytes located within the LPP plus UTP at P2, P6, P14, and 7dpp or bulge and secondary hair germ at 21dpp (complete data available in Table S1 and Fig. S2, S3, S4, S5, S6). KIT expression is reported as KIT^hi^ being melanocytes displaying high intensity fluorescence and KIT^+^ being melanocytes positive for KIT independent of fluorescence intensity. LPP, lower permanent portion of the hair; UTP, upper transitory portion of the hair; dpp, days post plucking; SHG, secondary hair germ of the hair; DP, dermal papilla; SG, sebaceous gland; Sc, subcutis.

Next we examined SOX10 expression in postnatal melanocytes during several key stages of hair morphogenesis and hair cycling (Fig. 1A); P2, P6, P14, adult anagen III/IV (7dpp) and adult catagen VII (21 days post plucking, 21dpp). Despite the availability of a number of useful fluorescent *Sox10* reporter mice [26]–[28], we opted to characterize protein expression with immunolabeling as this method is applicable to non-transgenic mouse studies. Using antibodies, we observed that nearly all DCT^+^ cells within hairs co-express SOX10 regardless of time point, location or differentiation status (Fig. S2).

We expanded our results for the McSC population by quantifying the percentage of DCT^+^/SOX10^+^ cells within the lower permanent portion (LPP) of the hair (hair bulge) and the upper transitory portion (UTP) of the hair since these regions contained the majority of DCT+ melanocytes that exist along the hair shaft (Fig. 1B, Table S1). As defined previously, the LPP extends from the opening of the sebaceous gland to the junction between the dermis and subcutis, and the UTP sits between the same junction and the hair bulb (Fig. 1A, [29]). In late catagen hairs (21dpp), the entire follicle exists within the dermis and is divided into the hair bulge and the secondary hair germ (SHG), with the SHG visible as a small cluster of cells between the hair bulge and dermal papilla (Fig. 1A, [30]). Between P2 and P14, SOX10 is detected in 94–99% of LPP+UTP melanocytes (Fig. 1B). SOX10^+^/DCT^+^ cells comprise 87% of the LPP+UTP melanocytes at anagen (7dpp), and 96% of bulge+SHG melanocytes at catagen (21dpp, Fig. 1B). This result contradicts previous reports showing that *Sox10* mRNA and protein are downregulated by the melanocytes that have colonized the hair bulge beginning at P2, and is absent in melanocytes found in catagen stage hairs [4], [31]. However, we suspect that higher sensitivity of our SOX10 antibody may limit our ability to distinguish melanocytes with variable levels of SOX10 expression, thus explaining our observation that DCT and SOX10 co-label more bulge melanocytes than previously reported.

In contrast to SOX10, the expression patterns of other melanocyte markers, MITF, KIT, TRP1 and TYR, within postnatal hairs is more variable (Fig. S3, S4, S5, S6). Beginning at P2, the majority of DCT^+^ LPP melanocytes in stage 4 hairs double-label with MITF and KIT whereas only 56% and 38% express the melanogenic enzymes TRP1 and TYR, respectively (Fig. 1B, Table S1). As hairs progress to stage 6 of their morphogenesis, nearly all LPP+UTP melanocytes continue to express MITF while the percentage of LPP+UTP melanocytes expressing TRP1 and TYR decreases. Previously, the intensity of KIT expression within the McSC niche was observed to be bipolar, either KIT^high/+^ or KIT^low/−^, with the KIT^low/−^ melanocytes corresponding to the McSC population [3], [32]. Although we rarely saw KIT^−^ melanocytes at any stage, we did observe an emergence of DCT^+^/KIT^low^ LPP+UTP cells in stage 6 hairs of P2 skins. By P14, LPP+UTP melanocytes have downregulated their differentiation markers and exist predominately in a SOX10^+^/MITF^+^/KIT^low^ state. Strikingly, the initiation of anagen (7dpp) corresponds with a dramatic escalation of the percentage KIT^high^ cells and a moderate increase of TRP1^+^ cells amongst LPP+UTP melanocytes. In contrast, entry into catagen (21dpp) is associated with LPP+UTP melanocytes downregulating MITF while still retaining TRP1, KIT and SOX10.

Across all hairs that contain bulbs (excluding catagen hairs), DCT^+^ melanocytes within the hair bulb also double-label with SOX10, MITF, TRP1 and TYR (Fig. S2, S3, S4, S5). KIT, on the other hand is robustly detected amongst melanocytes colonizing the bulbs of stage 4 morphogenetic hairs, but expressed with varying intensity in the matrix of older hairs (Fig. S6).

This analysis demonstrates that while the differentiation status of melanocytes that exists within the hair bulge fluctuates in concert with hair morphogenesis and adult hair cycling, SOX10 expression remains static amongst LPP and UTP melanocytes. We next assessed how perturbation of *Sox10* influences these expression patterns within follicular melanocytes.

### *Sox10* is required for the retention of McSCs and differentiated melanocytes and for pigment production

**Figure 2:**
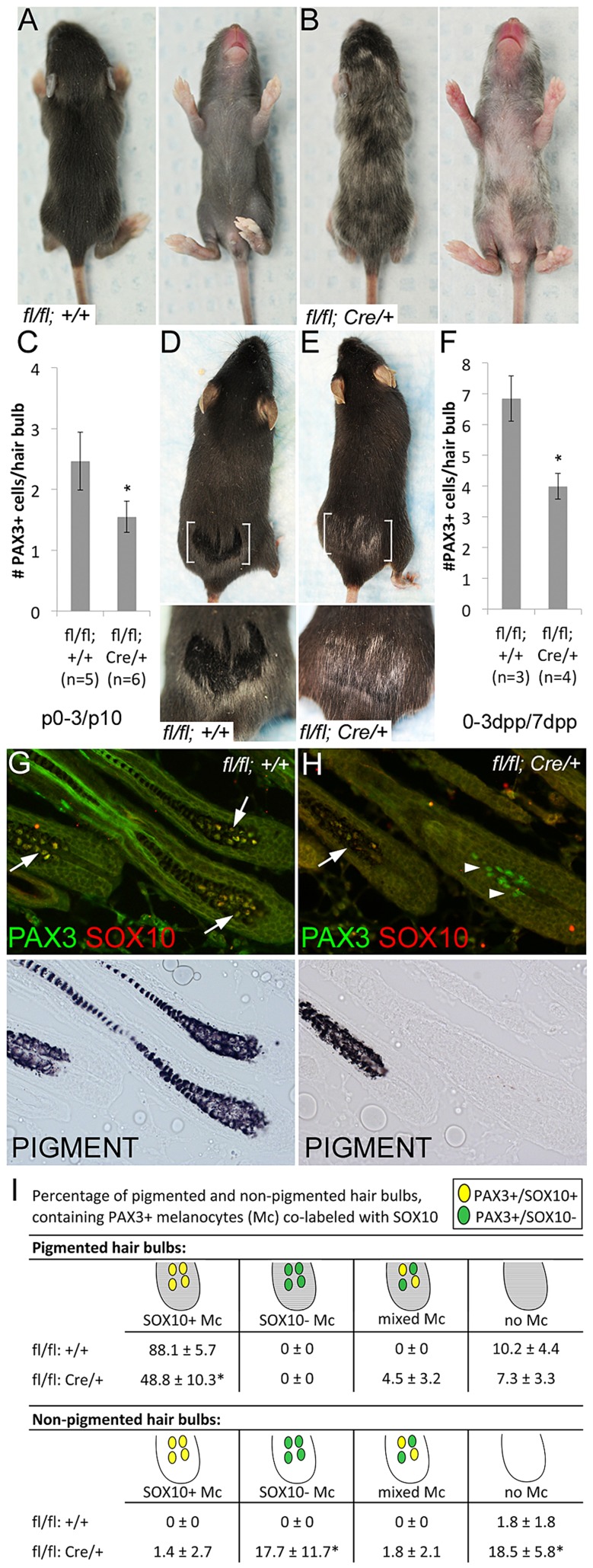
*Sox10* is required by bulb melanocytes postnatally. (A–B) *Sox10^fl/fl^* (*fl/fl; +/+*) and *Sox10^fl/fl^; Tyr::CreERT2* (*fl/fl; Cre/+*) pups treated with TAM by IP injection to the lactating mother on P0–3 display variegated hypopigmentation on the belly and back and exhibit a white head spot upon the emergence of the morphogenetic coat (P10 shown here, n>5). (C) Number of PAX3^+^ melanocytes per hair bulb in skins harvested from these mice at P10 are significantly decreased in *Sox10^fl/fl^; Tyr::CreERT2* animals compared to similarly-treated *Sox10^fl/fl^* animals (*p = 0.002). (D–E) Adult *Sox10^fl/fl^; Tyr::CreERT2* mice treated with TAM by IP injection on 0–3dpp exhibit white hairs within the plucked region upon hair regrowth that is not visible in similarly treated *Sox10^fl/fl^* mice (brackets indicate plucked region, lower image is a magnification of plucked region). (F) Number of PAX3^+^ melanocytes per hair bulb in skins harvested from similarly-treated mice at 7dpp are significantly decreased in *Sox10^fl/fl^; Tyr::CreERT2* animals compared to *Sox10^fl/fl^* animals (*p = 0.001). (G–H) Fluorescent and corresponding brightfield images of hair bulbs from mice described in D–E. Arrows and arrowheads indicate PAX3^+^/SOX10^+^ and PAX3^+^/SOX10^−^ melanocytes, respectively. (I) Distribution of melanocytes double-labeled for PAX3 and SOX10 within pigmented (gray) and non-pigmented (white) hair bulbs in skins from *Sox10^fl/fl^* (n = 3) and *Sox10^fl/fl^; Tyr::CreERT2* (n = 4) harvested on 7dpp from mice treated with TAM on 0–3dpp (*p<0.006).

In light of our discovery that SOX10 is retained by both McSCs and differentiated bulb melanocytes within the hair follicle, paired with previous in vitro experiments indicating that TYR expression in mouse is *Sox10-*dependent [15], we anticipate *Sox10* plays an important role in postnatal melanocyte biology. We tested this by generating *Sox10^fl/fl^; Tyr::CreERT2* mice to conditionally knockout *Sox10* in mouse melanocytes postnatally [33], [34]. Previously, we confirmed that *Tyr::CreERT2* is effective at inducing recombination of floxed alleles in a significant number of McSCs as well as the more differentiated melanocytes when TAM is administered transiently during perinatal growth or during adult anagen [25]. Using the same approach, we administered TAM in a pulse-like fashion to both pups and adult animals. Pups were given TAM just prior to their initial hair growth by receiving breastmilk from lactating mothers injected intraperitoneally (IP) with TAM on P0–P3. Adults, at approximately eight weeks of age, were plucked on their lower backs to induce anagen, and TAM was administered by IP injection on the same day as plucking and for three additional days (0–3dpp). In both cases, we observed hypopigmentation in a subset of the hairs in the *Sox10^fl/fl^; Tyr::CreER^T2^* mice in regions of the skin where newly grown hairs were emerging. This loss of pigmentation was not observed in similarly-treated *Sox10^fl/fl^* and *Sox10^fl/+^;Tyr::CreER^T2^* mice or *Sox10^fl/fl^; Tyr::CreER^T2^* mice that were not treated with tamoxifen (Fig. 2A–B, D–E; Fig. S7A–B). Using PAX3 as a marker for melanocytes, we found that this lack of pigmentation is associated with an overall reduction in the number of differentiated melanocytes per hair bulb, with a significant percentage of hair bulbs lacking melanocytes altogether (Fig. 2C, F). This indicates that *Sox10* is required for the retention of differentiated melanocytes in the hair. In *Sox10^fl/fl^; Tyr::*CreERT2 animals, we also observed a population of PAX3^+^/SOX10^−^ cells within the melanocytic region of the hair matrix whose presence correlated with hair bulbs that contained little or no pigmentation (Fig. 2G–I). This was particularly noticeable in TAM-treated *Sox10^fl/fl^; Tyr::*CreERT2 adults (Fig. 2I). This indicates that *Sox10* is also required by bulb melanocytes to differentiate, or produce pigment. These PAX3^+^/SOX10^−^ cells also do not express MITF, a SOX10 target gene, suggesting that the reduced pigment seen in SOX10^−^ bulb melanocytes is likely a result of aberrant melanocytic transcriptional regulation (Fig. S7C–D). The fate of melanocytes lacking *Sox10* remains unclear as positive staining for the apoptosis markers CC3 and/or TUNEL is not correlated with PAX3^+^/SOX10^−^ bulb cells or non-pigmented hairs in tamoxifen-treated *Sox10^fl/fl^; Tyr::*CreERT2 pups or adult animals (not shown). Nevertheless, these data demonstrate that the hypopigmentation observed with *Sox10* knockout is due to an overall loss of bulb melanocytes and a deficiency in their ability to produce pigment.

**Figure 3:**
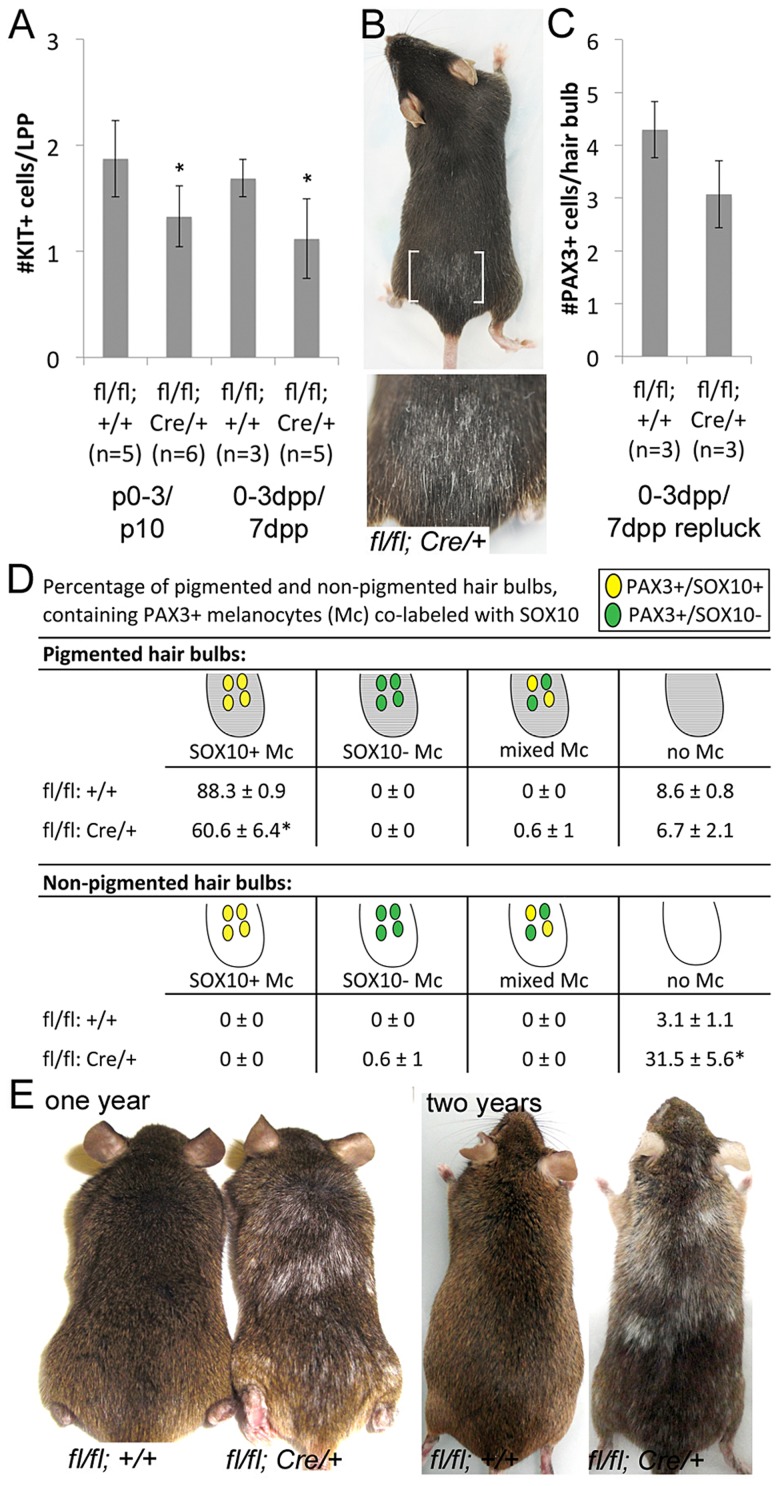
*Sox10* is required by LPP melanocytes postnatally. (A) Number of KIT^+^ LPP melanocytes within hairs from *Sox10^fl/fl^* (*fl/fl; +/+*) and *Sox10^fl/fl^; Tyr::CreERT2* (*fl/fl; Cre/+*) mice. P0–3/P10 indicates skins harvested from pups on P10 that were maintained by lactating mothers that were IP injected with TAM on P0–3. 0–3dpp/7dpp indicates skins harvested from adult mice on 7dpp after IP injections of TAM on 0–3dpp. (B) White hairs remain visible in adult *Sox10^fl/fl^; Tyr::CreERT2* mice that were treated with TAM by IP injection on 0–3dpp, allowed for complete hair regeneration, replucked and allowed for a second round of hair regrowth (brackets indicate plucked/replucked region, lower image is a magnification of plucked region; mouse in 2E and 3B are the same, imaged prior to and after replucking). (C) Number of PAX3^+^ bulb melanocytes within hairs from *Sox10^fl/fl^* and *Sox10^fl/fl^; Tyr::CreERT2* mice treated as described in B but harvested on 7dpp after replucking (0–3dpp/7dpp repluck). (D) Distribution of melanocytes double-labeled for PAX3 and SOX10 within pigmented (gray) and non-pigmented (white) hair bulbs in skins from *Sox10^fl/fl^* (n = 3) and *Sox10^fl/fl^; Tyr::CreERT2* (n = 3) mice treated as described in B but harvested on 7dpp after replucking (*p<0.002). (E) Persistent hair graying is visible in *Sox10^fl/fl^; Tyr::CreERT2* mice treated with IP TAM for pulse of five days beginning at five weeks old and imaged at one and two years old.

We have shown previously that the *Tyr::CreER^T2^* transgene is effective at inducing recombination in McSCs [25], and thus we also analyzed the effects of *Sox10* knockout on LPP (bulge) melanocytes. Using KIT as our marker for melanocytes, we discovered that LPP melanocytes are decreased in *Sox10^fl/fl^; Tyr::*CreERT2 mice when induced perinatally or as adults (Fig. 3A). Corroborating the idea that we are affecting the McSC population, we asked whether the white hairs and reduced LPP cells observed in TAM treated *Sox10^fl/fl^; Tyr::*CreERT2 adults are retained with hair cycling. To test this, we plucked adult animals on their lower back, administered TAM on 0–3dpp, allowed these hairs to regrow (similar to Fig. 2E), then replucked in the same region, and assessed this subsequent round of hair growth for pigmentation and melanocytes. The *Sox10^fl/fl^; Tyr::*CreERT2 mice treated in this manner still exhibit white hairs and reduced bulb melanocytes (Fig. 3B–C). However, the PAX3^+^/SOX10^−^ bulb cells that were observed in *Sox10^fl/fl^; Tyr::*CreERT2 mice after the initial adult treatment period (Fig. 2I) are rarely visible after replucking (Fig. 3D). Instead, the non-pigmented hairs in these replucked animals almost completely lack bulb melanocytes (Fig. 3D). This suggests that the PAX3^+^/SOX10^−^ bulb cells observed after the initial TAM treatment of *Sox10^fl/fl^; Tyr::*CreERT2 adults were likely a consequence of recombining the *Sox10^fl^* allele within a partially differentiated melanocyte rather than progeny from a PAX3^+^/SOX10^−^ McSC. Hypopigmentation observed in *Sox10^fl/fl^; Tyr::*CreERT2 animals treated with TAM at five weeks persists for at least two years of natural hair cycling (Fig. 3E), and together this data demonstrates that loss of *Sox10* leads to a permanent reduction in the number of McSCs and an inability of remaining McSCs to fully replenish the bulb melanocyte population in newly generated hairs.

Together these observations reveal a postnatal requirement for *Sox10* in mouse melanocytes. This extends to both the McSC and differentiated melanocyte populations and demonstrates that *Sox10* is necessary during the establishment of melanocytes within the hair follicle during hair morphogenesis as well as during the regeneration of melanocytes during adult hair cycling.

### Overexpression of *Sox10* induces McSC loss and premature hair graying

Expression of SOX10 by McSCs of the hair, a subpopulation that by their nature is inhibited from differentiation, suggests that McSCs uniquely regulate *Sox10* in order to maintain their stem cell properties. To determine whether changing the threshold of *Sox10* levels in the melanocyte lineage affects the ability of melanocytes to become established in the hair or maintained as McSCs, we examined mice that overexpress *Sox10* in melanocytes under the control of the *Dct* promoter (*Tg(DctSox10*, line CF1-10; [35]). This transgene exhibits a 2.4-fold increase in *Sox10* expression in skins obtained from *Tg(DctSox10)/+* animals compared to wild type (Fig. S8A).

**Figure 4:**
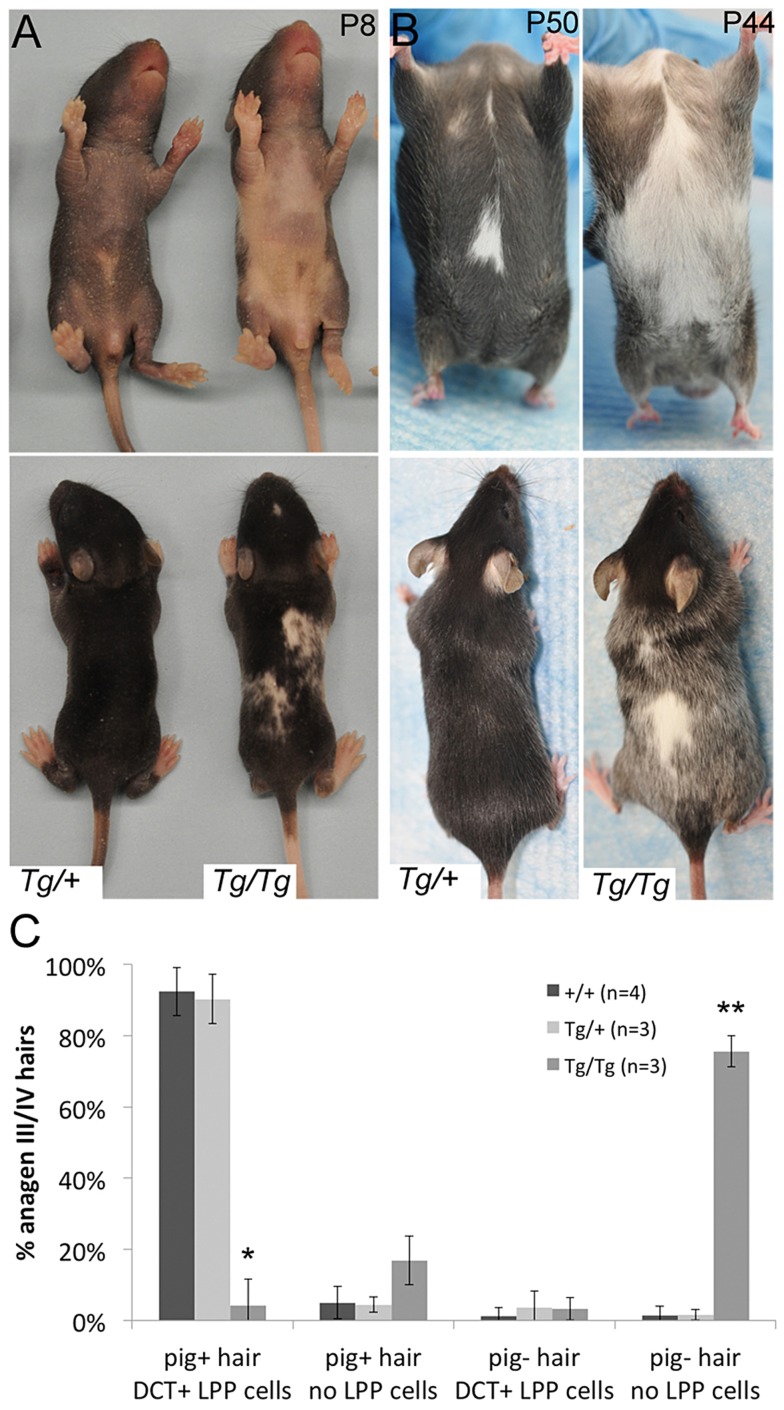
*Tg(DctSox10)* results in congenital white spotting and premature hair graying. (A, B) Ventral and dorsal views demonstrating variable hypopigmentation in *Tg(DctSox10)/+* and *Tg(DctSox10)/Tg(DctSox10)* mice during hair morphogenesis and adult hair cycling. (C) Frequency of pigmented (pig+) and non-pigmented (pig−) anagen III/IV (7dpp) hairs that contain (DCT+ LPP cells) or do not contain (no LPP cells) LPP melanocytes within *Tg(DctSox10)* or *+/+* mice. The ages of mice analyzed ranged between 9–22 weeks at harvest. Significance determined by chi-square analysis (p<<0.0001) and evaluation of standardized residuals (*, z = −8.84; **, z = 12.24).

The increase in *Sox10* expression manifests in two ways: congenital hypopigmentation (white spotting) and hair graying (Fig. 4A, B). At P8, *Tg(DctSox10)/+* mice exhibit hypopigmentation that is evident as small, ventral belly spots that are highly penetrant (97% in adults, n = 29/30 with belly spots; Fig. S8B). *Tg(DctSox10)/Tg(DctSox10)* mice at P8 have more extensive hypopigmentation with large white ventral spots that encompass the majority of the belly, dorsal spotting and occasional head spots. The white spotting observed with *Tg(DctSox10)* suggests that overexpression of *Sox10* affects the embryonic melanoblast population. *Tg(DctSox10)/Tg(DctSox10)* mice also display variable loss of hair pigmentation (premature hair graying) after the onset of the first adult hair cycle (first adult anagen is ∼P28, Fig. 4B).

Hair graying in *Tg(DctSox10)/Tg(DctSox10)* mice continues to increase progressively as these animals age (not shown). *Tg(DctSox10)/+* mice also exhibit hair graying but with a reduction in severity and with a later onset, beginning after the second adult hair cycle (second adult anagen is ∼12 weeks, 4/11 animals exhibit sparse gray hairs at ∼16 weeks). Hair graying in the *Tg(DctSox10)* line was first examined histologically in mice after the first adult hair cycle (between 9–22 weeks in age) and after hair cycle synchronization by plucking (Fig. 4C). Analysis at anagen (7dpp) demonstrated that in wild type and *Tg(DctSox10)/+* mice, the majority of hairs were both pigmented and contained LPP melanocytes (92.4±6.8% and 90.3±6.9%, respectively). In contrast, *Tg(DctSox10)/Tg(DctSox10)* mice exhibited primarily non-pigmented hairs that lacked LPP melanocytes (75.6±4.3%). From these observations we conclude that *Tg(DctSox10)*-induced hair graying is a direct consequence of McSC deficiency.

### Overexpression of *Sox10* disrupts McSC establishment

**Figure 5:**
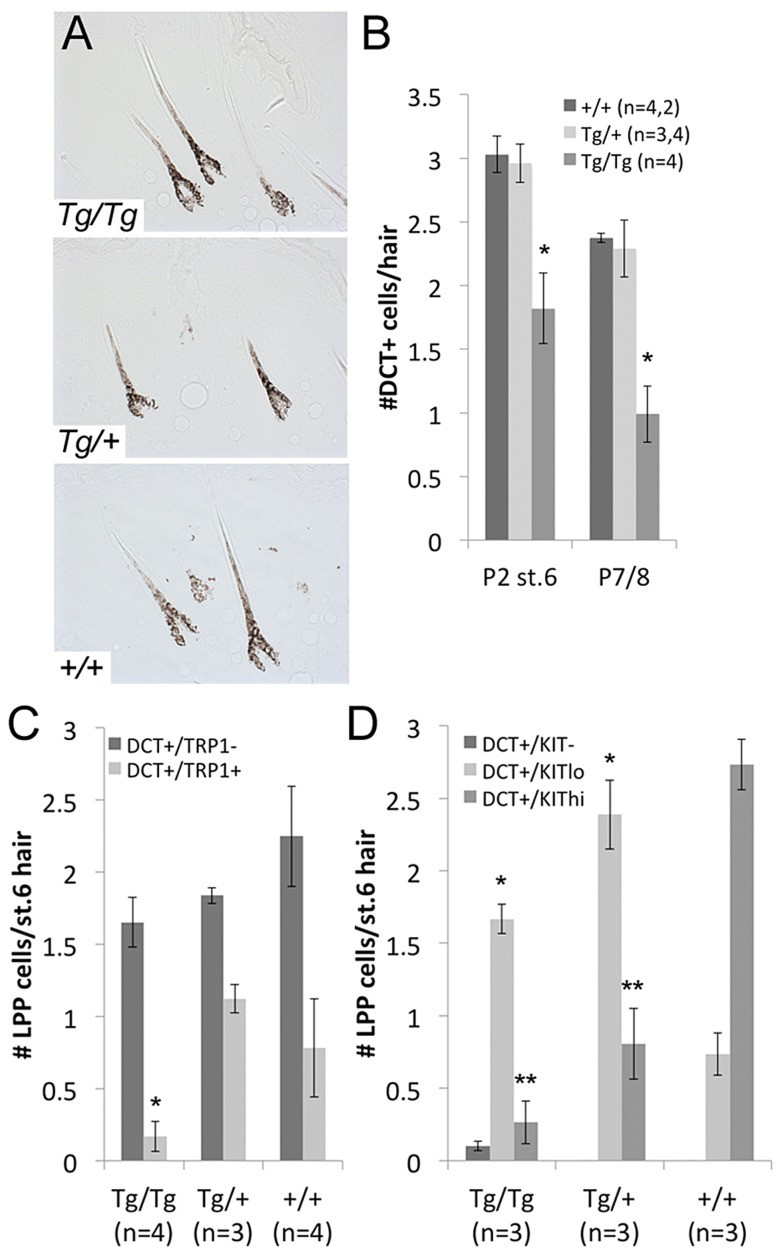
LPP melanocytes are reduced in *Tg(DctSox10)* homozygotes during hair morphogenesis. (A) Brightfield images of hairs in *Tg(DctSox10)* and *+/+* littermates at P2. (B) Number of DCT^+^ melanocytes within the LPP of hairs at P2 (stage 6 hairs) and P7/8. At both time points, LPP melanocytes per hair are reduced in *Tg(DctSox10)/Tg(DctSox10)* compared to *Tg(DctSox10)/+* and *+/+* mice (*p<0.017). (C, D) Quantitative immunohistochemical analysis of stage 6 hairs from P2 skins for DCT and TRP1, or DCT and KIT. The population of DCT^+^/TRP1^+^ cells is significantly reduced in *Tg(DctSox10)/Tg(DctSox10)* in comparison to *Tg(DctSox10)/+* and +/+ mice (*p<0.008). *Tg(DctSox10)* also causes a switch in KIT intensity from KIT^hi^ in wild type to KIT^low^ in *Tg(DctSox10)* animals (*KIT^lo^ and **KIT^hi^ comparisons made between +/+ and *Tg(DctSox10)/+* or *+/+* and *Tg(DctSox10)/Tg(DctSox10)*; p<0.005).

The fact that *Tg(DctSox10)/Tg(DctSox10)* animals exhibit premature hair graying at the first adult hair cycle suggests that the loss of McSCs observed in these animals occurs during hair morphogenesis when melanocytes colonize the hair. Melanocytes within the morphogenetic hair bulge and bulb are thought to become molecularly and anatomically distinct around P4 [4]. If *Sox10* overexpression affects McSC establishment then we would expect that the LPP melanocytes in *Tg(DctSox10)/Tg(DctSox10)* animals will decrease with age after P4. First, as indicated by the postnatal coat color, we confirmed in *Tg(DctSox10)* animals at P2 that regions of the coat unaffected by congenital white spotting contained hairs that were similarly pigmented in the hair bulb and shaft in comparison to *+/+* littermates (Fig. 5A). Counts of DCT^+^ cells within stage 6 hairs of P2 skins (the time point when the hair bulge is anatomically recognizable) show that while LPP melanocytes are detected in *Tg(DctSox10)/Tg(DctSox10)* mice, their numbers are moderately reduced. By P7/8, the number of LPP melanocytes in hairs of *Tg(DctSox10)/Tg(DctSox10)* mice decreases further to less than half of those observed in *Tg(DctSox10)/+* and *+/+* animals (Fig. 5B). The presence of a reduced number of LPP melanocytes in *Tg(DctSox10)* homozygotes confirms that while there may be fewer overall melanocytes in these animals due to the embryonic effects of *Sox10* overexpression, their ability to colonize the hair bulge at P2 is maintained. However, in *Tg(DctSox10)/Tg(DctSox10)* mice, the decrease in LPP melanocytes over time, and their absence in adults, suggests that high *Sox10* levels during melanocyte colonization of the hair follicle disrupts the establishment of McSCs.

To examine whether *Tg(DctSox10)* affects other melanogenic proteins within McSCs at P2, we evaluated the LPP expression patterns of TRP1, a known target of SOX10, and KIT receptor, and a protein not known to be downstream of SOX10. We find a noticeable depletion of DCT^+^/TRP1^+^ LPP melanocytes in *Tg(DctSox10)/Tg(DctSox10)* in comparison to *Tg(DctSox10)/+* or +/+ mice (Fig. 5C). We also find that *Tg(DctSox10)* dramatically changes the KIT expression profile; while the majority of LPP melanocytes are KIT^high^ in wild type, this switches to KIT^low^ in *Tg(DctSox10)* mice (Fig. 5D). Thus altered expression of TRP1 and KIT in LPP melanocytes precedes the loss of McSCs and consequent hair graying observed in *Tg(DctSox10)/Tg(DctSox10)* mice. Together these experiments demonstrate that although increased *Sox10* expression does not affect the ability of melanocytes to produce normally pigmented morphogenetic hairs, it does result in changes in the expression status of the LPP melanocytes that may affect their establishment as McSCs.

### Overexpression of *Sox10* disrupts McSC maintenance

**Figure 6:**
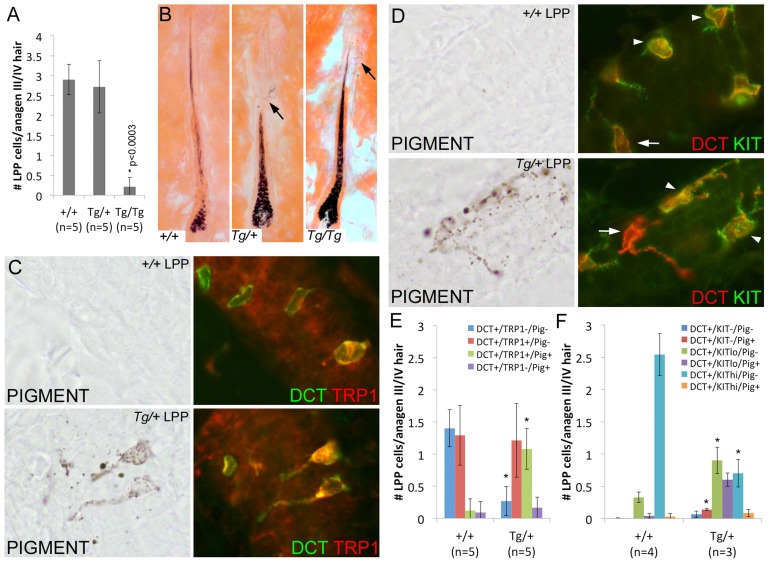
Overexpression of *Sox10* results in premature differentiation of LPP melanocytes in anagen hairs. (A) Number of DCT^+^ LPP melanocytes per anagen III/IV hair follicle (independent of the presence or absence of hair pigmentation) is significantly reduced in *Tg(DctSox10)/Tg(DctSox10)* mice when compared to wild type and *Tg(DctSox10)/+* mice (*p<0.0003). The ages of mice analyzed ranged between 9–22 weeks at harvest. (B) Eosin-stained skin sections of these hairs demonstrate the presence of ectopic pigmentation in the LPP of *Tg(DctSox10)/+* and *Tg(DctSox10)/Tg(DctSox10)* hairs (arrows) that is not see in wild type hairs. In *Tg(DctSox10)/+* LPP regions, this pigmentation often appeared in cells that were highly dendritic. (C, D) Brightfield and corresponding fluorescent images of anagen III/IV hair follicles double labeled for DCT and TRP1 (C) or KIT (D) in wild type and *Tg(DctSox10)/+* animals. The intensity of KIT fluorescence expression was variable, and categorized as KIT^lo^ (arrows) or KIT^hi^ (arrowheads), and did not appear to correlate with the presence or absence of pigmentation. (E,F) Comparison of the number LPP melanocytes per anagen III/IV hair follicle in *+/+* and *Tg(DctSox10)/+* animals that express DCT, and TRP1 or KIT, and produce ectopic pigmentation (*p<0.008).

The absence of McSCs in the adult hairs of *Tg(DctSox10)* homozygotes precludes our ability to phenotypically assess them at this age, however, the fact that *Tg(DctSox10)/+* mice exhibit changes in LPP expression profiles at P2 suggests that a closer look at *Tg(DctSox10)/+* skins is warranted. First as expected, immunolabeling validates the presence of SOX10 in both LPP and bulb melanocytes of anagen (7dpp) hairs in *Tg(DctSox10)/+* and wild type adult mice (Fig. S8C). Second, in contrast to the dramatic loss of LPP melanocytes observed in adult *Tg(DctSox10)* homozygotes, no change in the total number of melanocytes per LPP was detected within anagen (7dpp) hairs of *Tg(DctSox10)/+* animals in comparison to wild type (Fig. 6A) when assayed in mice ranging from 9–22 weeks of age.

Closer inspection of *Tg(DctSox10)/+* hairs revealed the presence of pigmented, often dendritic, cells within the McSC compartment. Ectopic LPP pigmentation was also detected in *Tg(DctSox10)/Tg(DctSox10)* hairs that remained pigmented into the adult hair cycle, but was rarely present in wild type hairs (Fig. 6B). LPP melanocytes of *Tg(DctSox10)/+* adult mice also exhibit changes in the expression pattern of TRP1 and KIT at anagen (Fig. 6C–F). In wild type animals, LPP melanocytes are mostly unpigmented and fall evenly into two categories, either DCT^+^-only or DCT^+^/TRP1^+^. In contrast, *Tg(DctSox10)/+* hairs contain considerably more pigmented DCT^+^/TRP1^+^ LPP melanocytes with an accompanying decrease in the number of DCT^+^-only LPP melanocytes (Fig. 6E). *Tg(DctSox10)* also affects the DCT/KIT expression profile in adult mice with *Tg(DctSox10)/+* hairs showing an increase in KIT^lo^/pigment^−^ and KIT^lo^/pigment^+^ LPP melanocytes at the expense of those that are KIT^hi^/pigment^−^ (Fig. 6F). These data indicate that increasing *Sox10* expression drives the inappropriate differentiation of LPP melanocytes into mature pigmented melanocytes. Together with the observation that *Tg(DctSox10)/+* animals also exhibit early hair graying we conclude that the *Sox10* levels must be tightly regulated to maintain the integrity of the McSC population.

### Changing levels of *Mitf* differentially affects congenital white spotting and hair graying in *Tg(DctSox10)* mice

*Sox10* is well documented as a transcription factor that binds directly to and regulates *Mitf*. In order to ascertain whether overexpression of *Sox10*, through an increase in *Mitf*, upregulates downstream pigment-producing genes, we asked whether reduction of *Mitf* could suppress pigmentation phenotypes in *Tg(DctSox10)*. Based on our observation that MITF is retained in the majority of LPP melanocytes at adult anagen (Fig. 1B), we first confirmed that a similar percentage of LPP cells in *Tg(DctSox10)/+* animals display normal MITF expression with approximately half exhibiting ectopic pigmentation (Fig. S8D–E).

**Figure 7:**
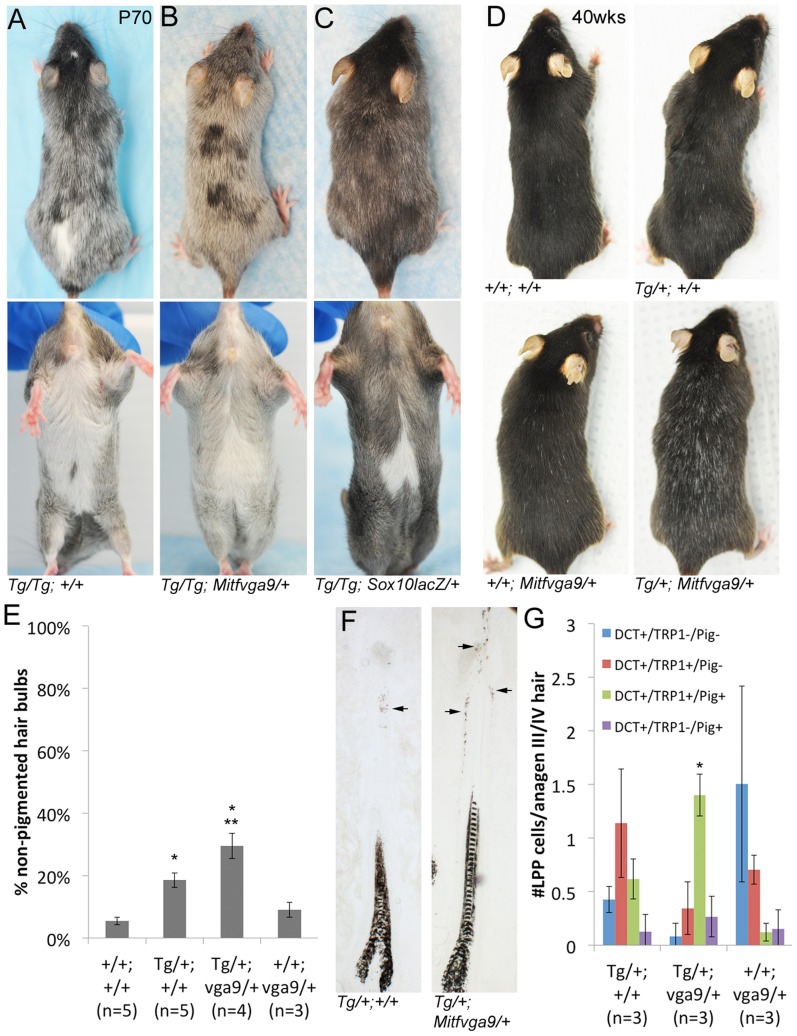
Alteration of the *Tg(DctSox10)* phenotype through the reduction of *Mitf*. (A–B) Comparison of *Tg(DctSox10)/Tg(DctSox10)* and *Tg(DctSox10)/Tg(DctSox10); Mitf^vga9/+^* animals at P70. Addition of the *Mitf^vga9/+^* allele reduces the congenital hypopigmentation seen in *Tg(DctSox10)/Tg(DctSox10)* animals, and is evident in dorsal views (loss of back spotting) and in ventral views (reduction in belly spot size). Premature hair graying of *Tg(DctSox10)* homozygotes seen at p70 is retained with *Mitf^vga9^* (n = 6). (C) Introduction of *Sox10^lacZ^* into *Tg(DctSox10)/Tg(DctSox10)* homozygotes partially rescues both congenital white spotting and premature hair graying (n = 2). (D) At 40 weeks of age, *Tg(DctSox10)/+; Mitf^vga9/+^* double heterozygotes exhibit visibly increased hair graying in comparison to *Tg(DctSox10)/+*. (E) Hair graying severity was determined in animals 6–10 weeks of age by quantitating the number of non-pigmented anagen III/IV hair bulbs in +/+, *Tg(DctSox10)/+, Tg(DctSox10)/+; Mitf^vga9/+^*, and *Mitf^vga9/+^* skins after plucking and harvesting at 7dpp. *Tg(DctSox10)/+; Mitf^vga9/+^* mice exhibit a significant increase in non-pigmented hair bulbs in comparison to the single heterozygotes or +/+ animals (**p<0.0015). *Tg(DctSox10)/+* animals also produce more non-pigmented hair bulbs in comparison to +/+ and *Mitf^vga9/+^* animals (*p<0.002). (F) *Tg(DctSox10)/+; Mitf^vga9/+^* animals (from E) display extensive ectopic pigmentation within the LPP of their hair follicles beyond what is normally observed in *Tg(DctSox10)/+* animals (arrows, n = 4). (G) Number of LPP melanocytes per anagen III/IV hair follicle in *Tg(DctSox10)/+, Tg(DctSox10)/+; Mitf^vga9/+^, Mitf^vga9/+^* animals (from E) that double label for DCT, TRP1, and produce ectopic pigmentation. Hairs from *Tg(DctSox10)/+; Mitf^vga9/+^* animals exhibit significantly more TRP1^+^/PIG^+^ LPP melanocytes than in either single heterozygote (*p<0.0125).

When mice that are *Tg(DctSox10)/Tg(DctSox10)* are combined with a hypomorphic *Mitf* mutant allele, *Mitf^vga9^* [36], they display a partial rescue of congenital hypopigmentation (normal dorsal pigmentation and a reduction in the size of ventral belly spotting). However, after the first adult hair cycle, these same *Tg(DctSox10)/Tg(DctSox10); Mitf^vga9/+^* mice proceed to gray prematurely like their *Tg(DctSox10)* homozygote counterparts (compare Fig. 7A, B). In contrast, we independently confirmed that both the congenital and graying phenotypes of *Tg(DctSox10)* are ameliorated by reducing endogenous *Sox10* with heterozygosity for a *Sox10* null allele (*Sox10^tm1Weg^*, here called *Sox10^lacZ^*; Fig. 7C). These data suggest that reduction of *Mitf* differentially affects the defects in embryonic melanoblasts and McSCs that are a result of increased *Sox10* expression.

Since LPP melanocytes of *Tg(DctSox10)* heterozygotes exhibit demonstrable changes in expression of differentiation markers, we evaluated whether *Mitf^vga9^* also affects this aspect of the *Sox10* overexpression phenotype. Upon visual inspection, an increased severity of hair graying in *Tg(DctSox10)/+; Mitf^vga9/+^* over *Tg(DctSox10)/+* mice is particularly noticeable as these animals approach one year of age (Fig. 7D, imaged at 40 weeks). Even by the second to third adult anagen (induced by plucking at 6–10 weeks of age), we observed significantly more non-pigmented hair bulbs in *Tg(DctSox10)/+; Mitf^vga9/+^* mice in comparison to either single heterozygote or *+/+* animals (Fig. 7E). Hairs from *Tg(DctSox10)/+; Mitf^vga9/+^* mice also display a noticeable expansion in the amount of ectopic pigmentation within the LPP in comparison to *Tg/+* animals (Fig. 7F). By immunofluorescent analysis we discovered that at this time point (6–10 weeks of age), nearly all melanocytes present in the LPPs of *Tg(DctSox10)/+; Mitf^vga9/+^* mice express TRP1 and are pigmented. This phenomena is less pronounced in *Tg(DctSox10)/+* mice and is not observed in *Mitf^vga9/+^* or *+/+* mice (Fig. 7G, 6E). The fact that loss of *Mitf* exacerbates rather than alleviates the premature differentiation of LPP melanocytes and hair graying seen in this *Tg(DctSox10)* line, suggests that MITF participates in the negative regulation of *Sox10* or *Sox10*-dependent processes within the McSC.

## Discussion

In this study we show that *Sox10* is critical postnatally for the establishment and maintenance of cells in the melanocyte lineage. The role of *Sox10* is twofold—first, it is necessary for the retention of mature bulb melanocytes and undifferentiated McSCs, and second, it is required for the production of normal follicular pigmentation. The apparently contradictory requirements for *Sox10* by undifferentiated McSCs and in the differentiation of melanocyte progenitors can be explained through our evidence that the McSC is maintained through the modulation of *Sox10* levels itself. Accordingly, while SOX10 is expressed at all stages of the melanocyte lineage in mouse, increased *Sox10* levels results in premature differentiation of the McSC population eliminating their capacity for self-renewal. This observation supports the idea that *Sox10* activity within the McSC is normally decreased, and we provide evidence suggesting that this may occur through a MITF-mediated negative feedback loop. From these observations we propose a model for McSC establishment during early postnatal development whereby melanocytes migrating into the morphogenetic hair assume either a stem or differentiated cell fate depending on the environment they colonize. In this model, the hair follicle bulge would activate unique stem cell-specific signaling pathways in resident melanocytes, including one involving MITF-mediated negative feedback. Subsequent downregulation of *Sox10* would establish the McSC. Melanocytes that colonize the hair bulb would not be subject to these repressive signals, *Sox10* activity would remain high, and these melanocytes would undergo differentiation. This mechanism is also applicable to the maintenance of the McSC and production of pigmented melanocytes during adult hair cycling.

The idea that *Sox10* can contribute to the preservation of the undifferentiated McSC population while also driving melanogenesis is in agreement with current views on the ability of SOX proteins to confer different states of cellular maturity. *Sox10*, in particular, is credited for defining successive stages of neural crest cell development during embryogenesis; beginning with the maintenance of multipotency within the neural crest stem cell [37], [38], and later for its participation in cell fate specification and survival of non-skeletogenic neural crest sublineages, particularly the melanocytes and glial cells (reviewed in [39]).

The mechanism by which *Sox10* levels are so precisely regulated within the postnatal melanocyte remains unclear. Previously, *Sox10* expression was reported to decrease as melanocytes colonized the hair bulge leading to the speculation that establishment of the McSC is dependent on downregulation of *Sox10* [4]. Despite the fact that our SOX10 immunolabeling does not exhibit the same temporal pattern, our loss and gain of function results do not contradict this theory. Basal levels of *Sox10* may provide survival of the postnatal melanocyte lineage, McSCs included, while a higher threshold of *Sox10* expression is required to drive melanocyte progenitor differentiation and pigment production. This idea is similar to the *Mitf* rheostat model proposed by Carreira et al. [40] to explain how varying levels of *Mitf* expression can produce a range of melanoma phenotypes from stem cell-like to proliferative to terminally differentiated. While the precise mechanisms regarding *Sox10* regulation are not fully known, conserved regulatory regions have been identified for *Sox10* and encompass binding sites for transcriptional activators including SOX9B, NOTCH, β-catenin, LEF1, MED1(PBP), ATF2, and TFAP2 [41]–[44]. WNT/β-catenin signaling, in particular, is a candidate for controlling the switch in *Sox10* expression—β-catenin remains in the cytoplasm of McSCs during telogen, but shuttles to the nucleus during anagen where it is sufficient to drive the melanocyte differentiation program [9]. Interestingly, constitutive activation of WNT signaling also results in ectopic pigmentation of McSCs and premature hair graying after several hair cycles.

The above observations do not discount the possibility that stem and progenitor fates in the melanocyte lineage may also be explained by a combinatorial mechanism where the availability of SOX10, regulatory regions of its targets, or partner transcription factors influence the cell state. For instance, SOX10 functions synergistically with a number of cofactors, namely PAX3, MITF, and CREB, during the activation of downstream genes [17], [45]–[48]. In particular, SOX10 and MITF cooperate to promote the transcription of DCT [17], [47], an interaction that is repressed by PAX3 and the corepressor GRG4. Displacement of PAX3 by activated β-catenin releases repression allowing SOX10/MITF-mediated upregulation of *Dct* expression [12]. Similar negative regulation of SOX10 function is observed in melanoblasts; SOX5 can both compete with SOX10 for binding while also recruit HDAC1 and CtBP2 corepressors to melanocyte gene promoters [20]. In other neural crest-derived cell types, the repression of SOX10 can also be achieved by direct sequestration. For example, in oligodendrocyte progenitors, an effector of Notch signaling, HES5, can bind SOX10 affecting its bioavailability [49]. This observation is intriguing in that *Hes1* and *Hes5* are expressed by melanoblasts, and that Notch signaling, is critical in their survival [50]. In particular, loss of Notch signaling in adult mice results in premature hair graying characterized by ectopically pigmented McSCs [29], [51], [52]. This suggests a possible link between the Notch pathway, *Sox10* and McSC maintenance.

A number of observations support the idea that MITF may repress McSC differentiation. First, the possibility of a negative feedback mechanism for the regulation of *Sox10* by MITF was shown using mathematical modeling to explain the dynamics of melanocyte differentiation within zebrafish [53]. Second, hypomorphic, *Mitf^vit^* ^/vit^ mice exhibit similar ectopic pigmentation and hair graying defects as we observed with *Tg(DctSox10)/+; Mitf^vga/+^* mice [8]. Lastly, the fact that *Mitf^vga9^* reduces congenital white spotting but exacerbates hair graying in with *Tg(DctSox10)* suggests a role for MITF within the McSC that is unique from its role within the melanoblast. In regards to the latter, we believe the white spotting phenotypes observed in *Tg(DctSox10)* mice may be explained by increased *Mitf* expression. MITF directly binds and upregulates genes required for melanin synthesis and melanosome biogenesis, including *Tyr*, *Trp1*, *Dct*, *SILV*, *MLANA*, and *GPR143* [12], [54]–[57]. MITF is also widely implicated in cell cycle regulation. In particular, MITF can positively control transcription of the cell cycle inhibitor genes, *CDKN1A* (p21) and *CDKN2A* (p16) [58], [59]. Fittingly, loss of MITF results in increased proliferation of melanoblasts in vivo [60]. Studies of the *Chx10* mutant mouse reveal that inappropriate maintenance of *Mitf* within the retinal progenitor cells leads to their reduced proliferation, transdifferentiation into pigmented cells, and consequent micropthalmia [61], [62]. Anecdotally, we observe that *Tg(DctSox10)/Tg(DctSox10)* mice have small eyes that are rescued by haploinsuffiency for *Mitf* (Hakami, RM, Arnheiter, H, and Pavan WJ; unpublished observation). Together these observations indirectly support the idea that the hypopigmentation observed in this *Tg(DctSox10)* line may be attributed to increased levels of MITF within melanoblasts inhibiting their proliferation and/or causing their inappropriate temporal differentiation.

The presence of ectopically pigmented cells within the hair bulges of *Tg(DctSox10)* mice fits with the assertion that overexpression of Sox10 drives the premature differentiation of McSCs. The increase in the percentage of LPP melanocytes that are TRP1^+^/pigment^+^ in hairs of adult *Tg(DctSox10)* heterozygotes compared to wild type animals confirms this. However, we also observed an unexpected change in KIT receptor expression in LPP melanocytes with *Sox10* overexpression. At adult anagen, the majority of bulge melanocytes in wild type mice exhibit high KIT immunofluorescence intensity (KIT^hi^) and those in *Tg(DctSox10)* mice appear KIT^low^. Previous reports show that McSC progenitors rely on KIT signaling for their appropriate proliferation and pigmentation during hair growth, and bulge melanocytes that retain a KIT^low/−^ status represent the McSC population. [2], [6], [32]. Together with our data, showing that overexpression of *Sox10* produces numerous pigmented, Kit^low^ bulge melanocytes, suggests that regulation of melanocyte lineage differentiation can also occur independent of high KIT expression. This idea is supported by the observation that *Kit* mutants, when treated with ionizing radiation, produce ectopic pigmentation within the hair bulge and exhibit hair graying [63]. No evidence to date has identified a role for SOX10 in the transcriptional control of *Kit*, and this is exemplified in recent microarray studies showing that *Sox10* knockdown in melanoma cells results in no significant change in *Kit* expression (data analysis by GEO2R for datasets GSE37059, GSE25501; [64], [65]). Further investigation into *Kit* regulation and how KIT signaling contributes to McSC maintenance during aging is warranted.

The translational importance of *Sox10* in melanocytic disease is highlighted in recent studies linking *Sox10* with cell cycle regulation and reduction of SOX10 expression correlating with reduced tumor cell burden in a mouse melanoma model [64]. Our study on the role of *Sox10* in the postnatal follicular melanocytes suggests a mechanism where SOX10 supports the maintenance of the melanocyte lineage while being inhibited from driving McSC differentiation. Our illustration of how tissue-specific stem cells might arise from lineage-specified precursors, and how this can occur through the regulation of the transcription factors critical in specifying this lineage may lead to further insights into how these processes can be disrupted or manipulated within disease.

## Materials and Methods

### Ethics statement

Animal care and experimental animal procedures were performed in accordance with the NIH IACUC.

### Animals

*TYR::CreER^T2^* and *Sox10^LacZ^* (*Sox10^tm1Weg^*) mice were rederived on and maintained by outcross to C57BL/6J [34], [66]. *Rosa26^tm1sor^* mice were obtained as homozygotes, maintained by intercross and bred together with *TYR::CreER^T2^* mice to generate compound heterozygotes [67]. *Sox10^fl^* and *Mitf^vga9^* mice were rederived on C57BL/6J and maintained by intercross [33], [68]. The *Tg(DctSox10)* line (CF1-10, [35]) was maintained through a combination of outcrossing to C57BL/6J and by intercross.

### Genotyping

Mice were genotyped using DNA isolated from tail tips and PCR analysis. Primers for the *TYR::CreER^T2^* allele, 5′-TCCGCCGCATAACCAGTGAA-3′ and 5′- CGGAAATGGTTTCCCGCAGA, were used to amplify the *Cre recombinase* sequence under standard PCR conditions (30 cycles of 45 s at 94°C, 45 s at 65°C and 60 s at 72°C). *Mitf^vga9^* and *Sox10^LacZ^* alleles were detected using PCR primers for β-galactosidase, 5′-GATCCGCGCTGGCTACCGGC-3′ and 5′-GGATACTGACGAAACGCCTGCC-3′, using the same PCR conditions described above. Primers and cycling conditions for the *Sox10^fl^* allele was described previously [33]. Zygosity for the *Tg(DctSox10)* transgene was determined by TaqMan analysis for two SNPs flanking the transgene on chromosome 1 that distinguish the original FVB donor strain from the C57BL/6 background strain (rs13475895 and rs13475987).

### Induction of CRE activity

TAM (T5648, Sigma) was dissolved in corn oil or a combination of ethanol and sunflower oil. TAM treatment was performed by IP injection of lactating mothers or adults with 2 mg/animal for the number of days indicated.

### Hair cycle staging and synchronization

Morphogenetic and adult hairs were staged according to [69], [70]. Plucking was performed to synchronize adult hairs. Briefly, mice were anesthetized and hairs were removed by hand over a 1.5 cm×2 cm region on the lower back. Hairs within this region were allowed to regenerate for 7 (7days post plucking, 7dpp) or 21 days (21 days post plucking, 21dpp). At each stage, the regions of the hair follicle were strictly defined based on visible anatomical landmarks (as described in [29]).

### Immunohistochemistry

After shaving, skin from the lower back was immersed in 2% formaldehyde, and irradiated in a 540W variable wattage microwave (BioWave, Pelco) three times in intervals of 30 s irradiation followed by 60 s on ice. After microwaving, samples remained in fixative for an additional 25 minutes on ice. Skins were cryoprotected in 10% sucrose overnight, embedded in NEG-50 (Thermo Scientific), frozen and sectioned with a cryostat (10 µm). For brightfield imaging, eosin-Y was sometimes used as a counterstain.

Sections for immunolabeling were first rinsed in PBS with 0.1% Tween 20. For nuclear antigens sections were subjected to antigen retrieval by boiling for 20 minutes in a Tris-EDTA solution and then permeabilized by treating with 1% Triton X-100 for 15 minutes. Sections were blocked for two hours in 1% bovine serum albumin (Sigma) and incubated with primary antibody overnight at 4°C. Primary antibodies include those against DCT (1∶300; TRP2, Santa Cruz Bio, sc-10451), SOX10 (1∶75; Santa Cruz Bio, sc-17342), PAX3 (1∶75, Developmental Studies Hybridoma Bank), MITF (1∶1000; rabbit polyclonal, gift from Heinz Arnheiter, NINDS-NIH), c-KIT (1∶100; ACK4, Cedarlane, CL8936AP), TRP1 and TYR (1∶300; PEP-1 and PEP-7, rabbit polyclonals, gift from Vince Hearing, NCI-NIH), Cre recombinase (1∶1000; Novagen, #69050-3), β-galactosidase (1∶32,000; MP Bio, #08559761), and cleaved Caspase-3 (1∶100; Cell Signaling, #9661). After washing, sections were incubated in the appropriate secondary antibodies (1∶5000; Alexafluor488 or 568, Invitrogen) for two hours at room temperature.

Sequential immunolabeling was performed for co-detection of DCT and SOX10 as these antibodies were both generated in goat. After labeling for SOX10 using the protocol described above, sections were blocked with rabbit α-goat IgG FAB (1∶10; Jackson Immuno, #305-007-003) for two hours, washed and then labeled for DCT as described above.

Brightfield and fluorescence microscopy was performed on a Zeiss Observer.D1 compound microscope. Images were obtained with an Axiocam Hrc camera using the Axiovision 4.8.2 software and processed with Adobe Photoshop. Quantitation of hair and cell phenotypes of immunolabeled tissue was performed on every fourth section of sequentially obtained skin sections. Data is presented as the mean ± standard deviation. Student's T-test with Bonferroni correction was used to determine statistical significance, unless stated otherwise.

### β-galactosidase staining

Skin samples were fixed in 2% formaldehyde/0.2% glutaraldehyde for 30 min at room temperature. Samples were then washed with rinse buffer (2 mM MgCl2/0.1% NP40/PBS) and stained overnight in X-Gal solution consisting of 0.32 mg/ml X-Gal, 5 mM ferrothiocyanide, and 5 mM ferrithiocyanide in rinse buffer.

### Quantitative PCR

RNA from E17.5 skins from wild type and *Tg(DctSox10)/+* mice was reverse transcribed using the High Capacity cDNA Reverse Transcription Kit (ABI). Quantitative PCR was performed using Taqman Fast Universal PCR Master Mix (ABI) and the following Taqman gene expression assays: Sox10 (Mm01300162_m1) and Pax3 (Mm00435493_m1). All experiments were performed with technical and biological replicates of ≥3.

## Supporting Information

Figure S1 Perinatal melanocytes give rise to the McSC population. (A) Anti-Cre recombinase (green) is present in the majority of DCT^+^ melanocytes (red) present in the skin at P2. (B, C) Skins from *Tyr::CreER^T2^; Rosa26^tm1sor^* reporter mice treated at P2 and P3 with tamoxifen were harvested and analyzed for Bgal (blue) activity at P14 and 7 days post plucking (7dpp). Bgal^+^ cells are visible in the LPP (arrows) and bulb of the hair at both timepoints confirming that induction of *Tyr::CreER^T2^* perinatally successfully targets McSCs and their more differentiated progeny. (D) Double immunolabeling of these 7dpp skins reveals that 97% of perinatally lineage-marked Bgal^+^ cells (green) that exist with the LPP are DCT^+^ melanocytes (red, arrows; 115 LPP cells analyzed across 3 animals).
(TIF)

Figure S2 SOX10 expression in follicular melanocytes. Immunofluorescence staining of skins harvested at P2, P6, P14, 7dpp and 21 dpp reveals that the majority of DCT^+^ melanocytes (melanosomal, red) located in the LPP and bulb of the hair follicle also express SOX10 (nuclear, green). Arrows indicate examples of double-labeled cells.
(TIF)

Figure S3 MITF expression in follicular melanocytes. Immunofluorescence staining of skins harvested at P2, P6, P14, 7dpp, and 21dpp for DCT (melanosomal, red) and MITF (nuclear, green). Double-labeled cells are apparent in the LPP and bulb of the hair from P2 through 7dpp, but are not visible in melanocytes at 21dpp. Arrows indicate examples of double-labeled cells.
(TIF)

Figure S4 TRP1 expression in follicular melanocytes. Immunofluorescence staining of skins harvested at P2, P6, P14, 7dpp, and 21dpp for DCT (melanosomal, red) and TRP1 (melanosomal, green). TRP1 expression is visible in hair bulb melanocytes throughout hair cycling, but is variable in LPP melanocytes. At P6 very few LPP melanocytes express TRP1, but this number increases through P14 and 7dpp and then remains relatively static during 21dpp. Arrows indicate examples of double-labeled cells.
(TIF)

Figure S5 TYR expression in follicular melanocytes. Immunofluorescence staining of skins harvested at P2, P6, P14, 7dpp, and 21dpp for DCT (melanosomal, red) and TYR (melanosomal, green). In general TYR expression is detected most strongly in the melanocytes that exist in the hair bulb, and very rarely in LPP melanocytes. Few TYR^+^ melanocytes are detected at catagen, shown at 21dpp. Arrows indicate examples of double-labeled cells.
(TIF)

Figure S6 KIT expression in follicular melanocytes. Immunofluorescence staining of skins harvested at P2, P6, P14, 7dpp, and 21dpp for DCT (melanosomal, red) and KIT (membrane-bound, green). In the LPP of hairs at P2, P6, P14 and 7dpp nearly all melanocytes are KIT^+^, but with variable fluorescence signal intensity. At 7dpp, KIT highlights the dendricity of some LPP melanocytes. In the bulbs of P6, P14 and 7dpp hairs KIT expression is strongly localized to the keratinocytes at the bulb tip (previously reported, [71]), but is also apparent in a more diffuse, speckled pattern in the hair matrix where the differentiated melanocytes exist. KIT expression is also retained in nearly all melanocytes through catagen, shown at 21dpp. Arrows indicate examples of double-labeled cells.
(TIF)

Figure S7 *Sox10* loss results in a corresponding loss in MITF expression. (A–B) Control animals do not exhibit a hypopigmentation phenotype; (A) *Sox10^fl/+^; Tyr::CreERT2* animal treated with topical tamoxifen at P2–3 and imaged at P14, (B) untreated *Sox10^fl/fl^; Tyr::CreERT2* animal imaged at P43. (C) Triple-labeling of hair bulbs from *Sox10^fl/fl^; Tyr::CreERT2* (*fl/fl; Cre/+*) mice described in Fig. 2E. Arrows and arrowheads indicate PAX3^+^/MITF^+^/SOX10^+^ and PAX3^+^/MITF^−^/SOX10^−^ melanocytes, respectively. (D) Distribution of melanocytes double-labeled for PAX3 and MITF within pigmented (gray) and non-pigmented (white) hair bulbs in skins from *Sox10^fl/fl^* (*fl/fl; +/+*, n = 3) and *Sox10^fl/fl^; Tyr::CreERT2* (*fl/fl; Cre/+*; n = 4) harvested on 7dpp from mice treated with TAM on 0–3dpp (*p<0.0083).
(TIF)

Figure S8 *Tg(DctSox10)* results in an increase in *Sox10* expression, and belly spotting, but no change in the MITF expression profile of LPP melanocytes. (A) Quantitative RT-PCR of e17.5 back skin confirms that *Tg(DctSox10)/+* mice exhibit a 2.4-fold increase in *Sox10* mRNA levels in comparison to wild type when normalized to *Pax3* (p<0.001, Student's t-test). *Pax3* was used for normalization to control for possible variation in melanoblast numbers between genotypes. (B) The white belly spot observed in *Tg(DctSox10)/+* mice varied in size from 0–10 white hairs up to a white spot measuring 7×15 mm. The penetrance of the white belly spot in *Tg(DctSox10)/+* mice was 97% (29/30). Occasionally, a few white belly hairs were also observed in the background strain, C57Bl/6 (15%, 4/27). (C) SOX10 (green) is evident within the LPP and bulb melanocytes (DCT^+^, red) of both *+/+* and *Tg(DctSox10)/+* mice. (D) Brightfield and corresponding fluorescent images of anagen III/IV hair follicles double labeled for DCT and MITF in wild type and *Tg(DctSox10)/+* animals. (E) Comparison of the number LPP melanocytes per anagen III/IV hair follicle in *+/+* and *Tg(DctSox10)/+* animals that express DCT, and MITF, and produce ectopic pigmentation (*p<0.0001). The average number of MITF^+^ melanocytes per LPP was not significantly different between *Tg/+* (2.81±0.97) and wild type (2.78±0.19) animals (p = 0.96).
(TIF)

Table S1 Quantitation of melanocyte immunolabeling during hair morphogenesis and hair cycling. Percentage of DCT^+^ melanocytes doubled labeled with the indicated marker per LPP+UTP or Bulge+SHG (∼25–50 hairs analyzed/animal, n = 3 animals per timepoint, data reported as mean ± S.D.). Grayed cells indicate the combination with the highest percentage of cells doublelabeled. *SOX10 expression in melanocytes at 21dpp is present, but weak, and normal staining protocols for DCT/SOX10 double labeling diminished visible SOX10 signal. Thus melanocytes at this timepoint were identified using KIT and then double labeled for SOX10. LPP, lower permanent portion of the hair; UTP, upper transitory portion of the hair; dpp, days post plucking; SHG, secondary hair germ of the hair.
(PDF)
