# Supplementary figures and images for: A Dual Role for SOX10 in the Maintenance of the Postnatal Melanocyte Lineage and the Differentiation of Melanocyte Stem Cell Progenitors
Source: PLoS Genet. 2013 Jul 25;9(7):e1003644. doi: 10.1371/journal.pgen.1003644 (PMC3723529; doi:10.1371/journal.pgen.1003644)

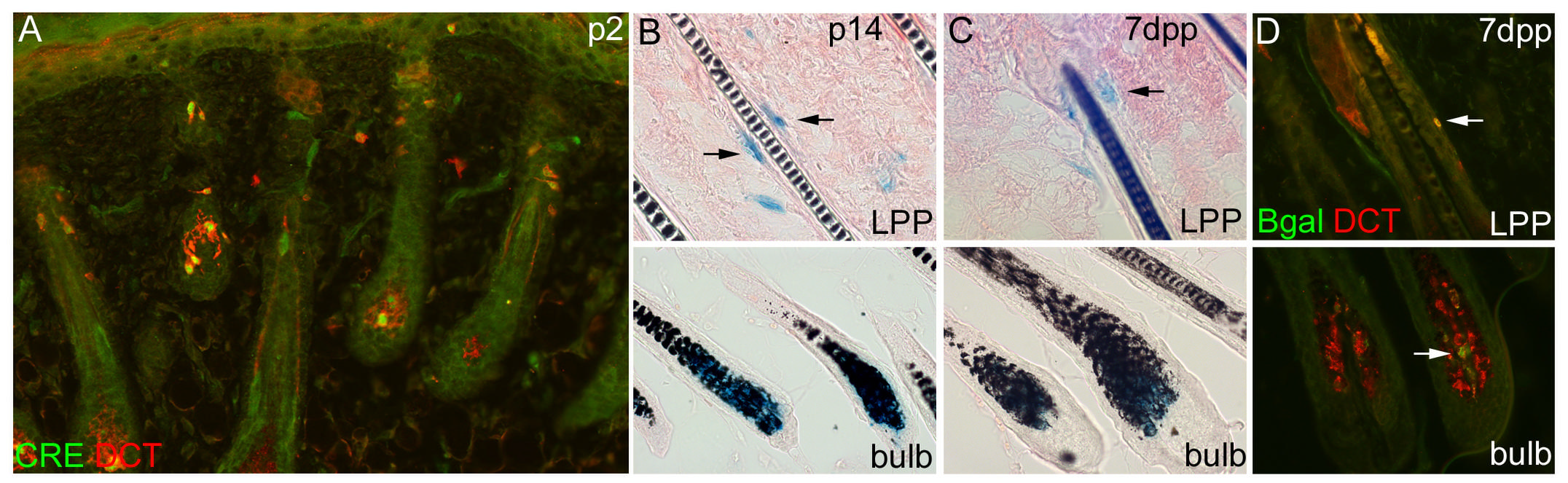

Supplement: Figure S1 — Perinatal melanocytes give rise to the McSC population. (A) Anti-Cre recombinase (green) is present in the majority of DCT+ melanocytes (red) present in the skin at P2. (B, C) Skins from Tyr::CreERT2; Rosa26tm1sor reporter mice treated at P2 and P3 with tamoxifen were harvested and analyzed for Bgal (blue) activity at P14 and 7 days post plucking (7dpp). Bgal+ cells are visible in the LPP (arrows) and bulb of the hair at both timepoints confirming that induction of Tyr::CreERT2 perinatally successfully targets McSCs and their more differentiated progeny. (D) Double immunolabeling of these 7dpp skins reveals that 97% of perinatally lineage-marked Bgal+ cells (green) that exist with the LPP are DCT+ melanocytes (red, arrows; 115 LPP cells analyzed across 3 animals). (TIF) [file pgen.1003644.s001.tif]

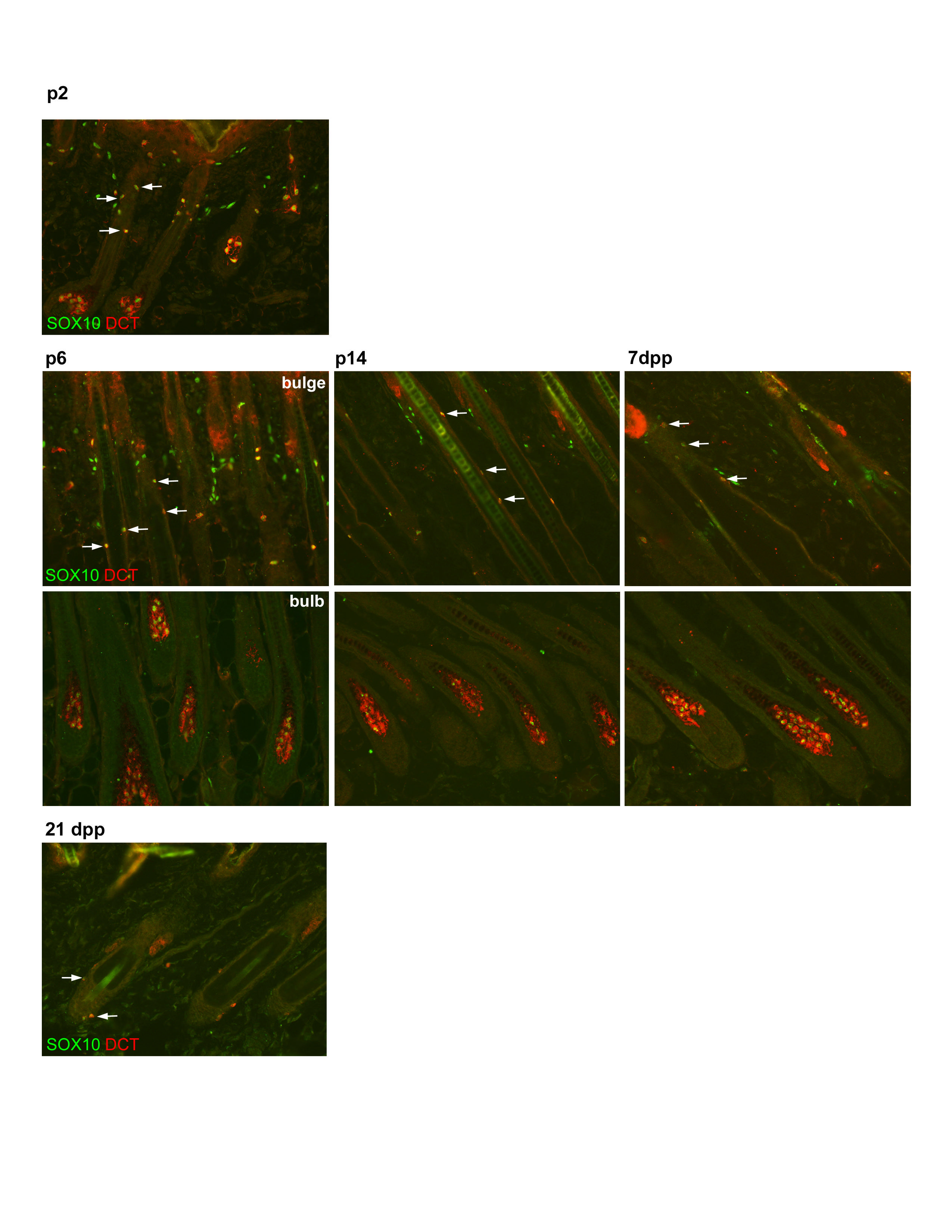

Supplement: Figure S2 — SOX10 expression in follicular melanocytes. Immunofluorescence staining of skins harvested at P2, P6, P14, 7dpp and 21 dpp reveals that the majority of DCT+ melanocytes (melanosomal, red) located in the LPP and bulb of the hair follicle also express SOX10 (nuclear, green). Arrows indicate examples of double-labeled cells. (TIF) [file pgen.1003644.s002.tif]

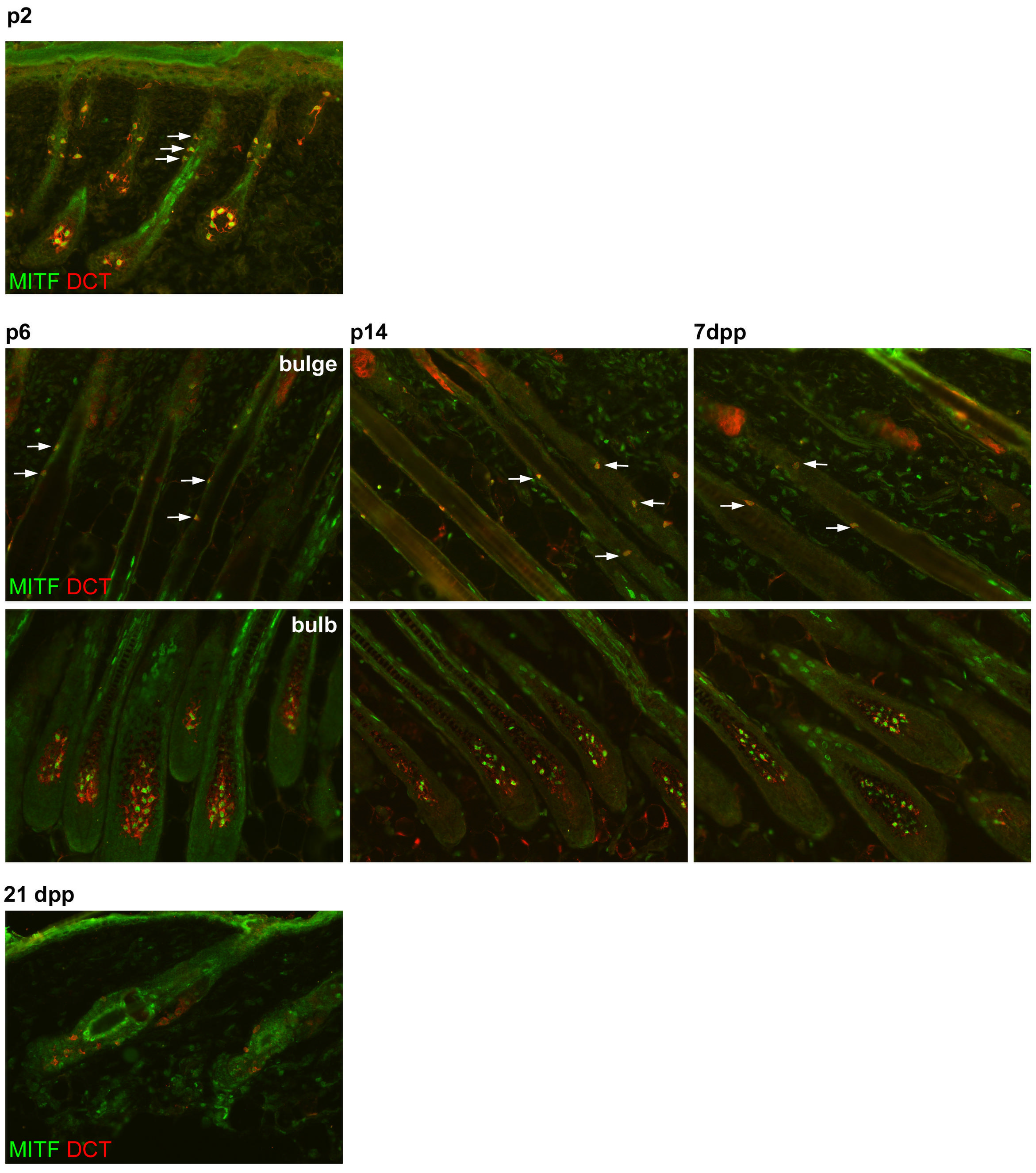

Supplement: Figure S3 — MITF expression in follicular melanocytes. Immunofluorescence staining of skins harvested at P2, P6, P14, 7dpp, and 21dpp for DCT (melanosomal, red) and MITF (nuclear, green). Double-labeled cells are apparent in the LPP and bulb of the hair from P2 through 7dpp, but are not visible in melanocytes at 21dpp. Arrows indicate examples of double-labeled cells. (TIF) [file pgen.1003644.s003.tif]

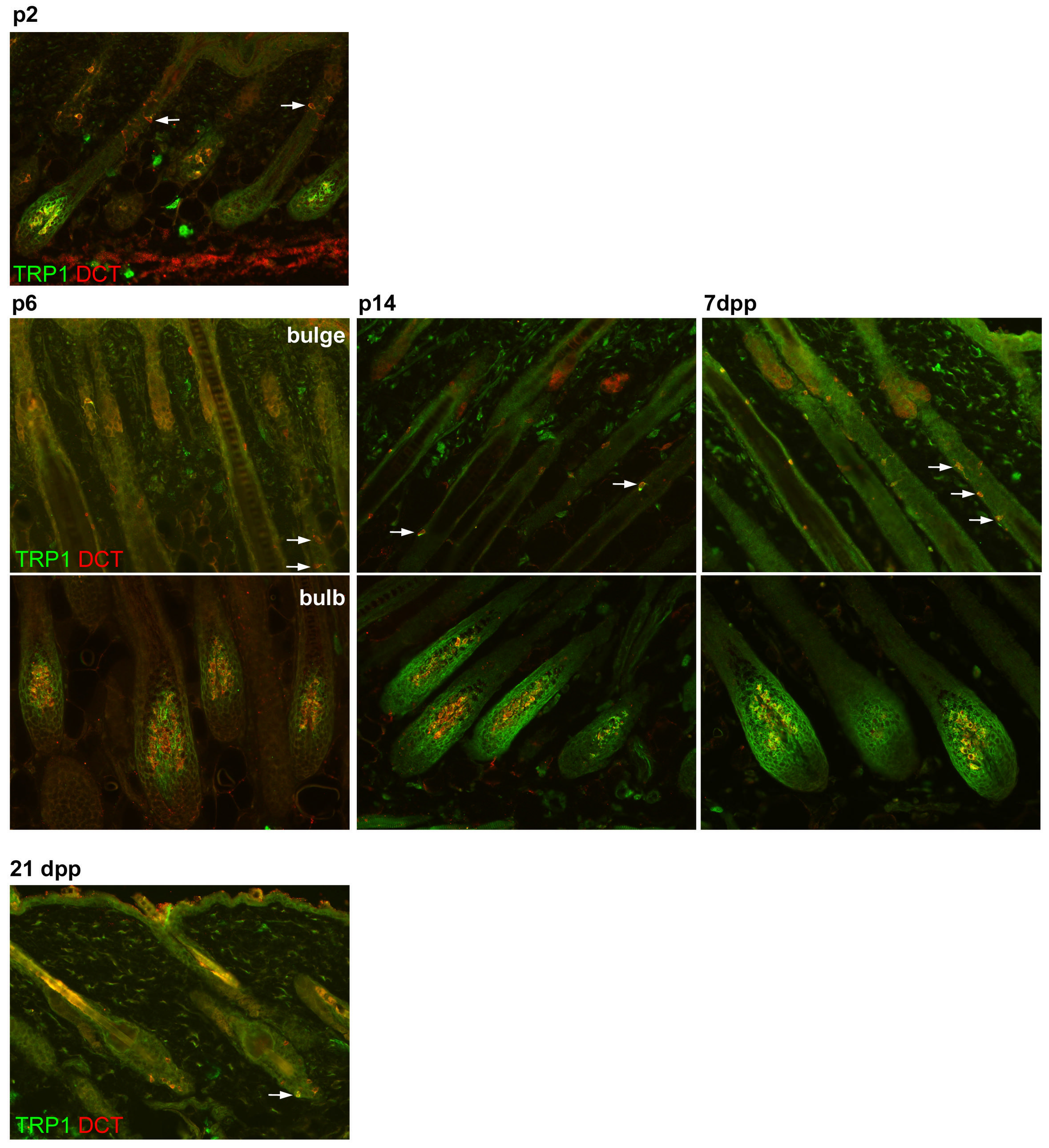

Supplement: Figure S4 — TRP1 expression in follicular melanocytes. Immunofluorescence staining of skins harvested at P2, P6, P14, 7dpp, and 21dpp for DCT (melanosomal, red) and TRP1 (melanosomal, green). TRP1 expression is visible in hair bulb melanocytes throughout hair cycling, but is variable in LPP melanocytes. At P6 very few LPP melanocytes express TRP1, but this number increases through P14 and 7dpp and then remains relatively static during 21dpp. Arrows indicate examples of double-labeled cells. (TIF) [file pgen.1003644.s004.tif]

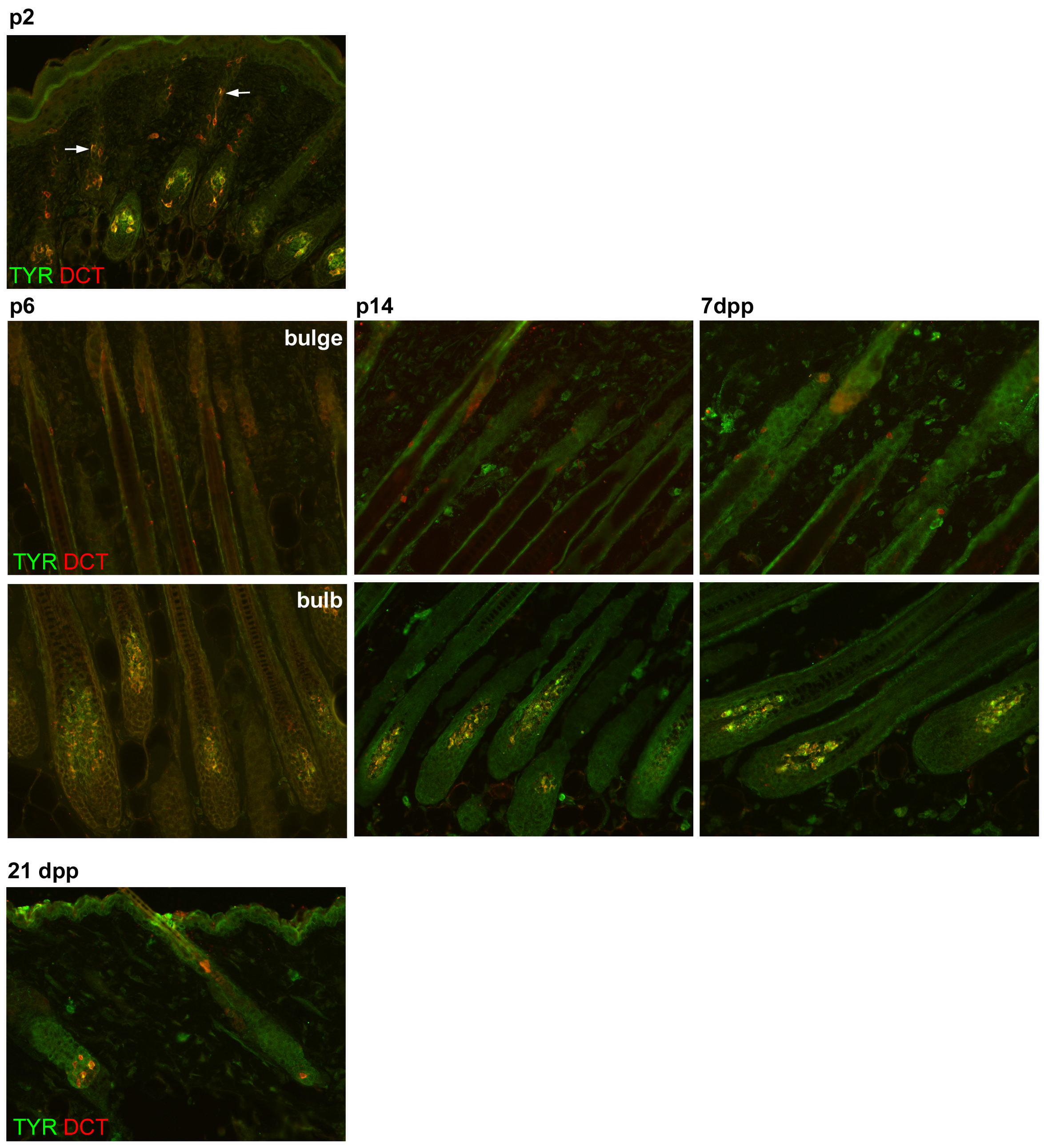

Supplement: Figure S5 — TYR expression in follicular melanocytes. Immunofluorescence staining of skins harvested at P2, P6, P14, 7dpp, and 21dpp for DCT (melanosomal, red) and TYR (melanosomal, green). In general TYR expression is detected most strongly in the melanocytes that exist in the hair bulb, and very rarely in LPP melanocytes. Few TYR+ melanocytes are detected at catagen, shown at 21dpp. Arrows indicate examples of double-labeled cells. (TIF) [file pgen.1003644.s005.tif]

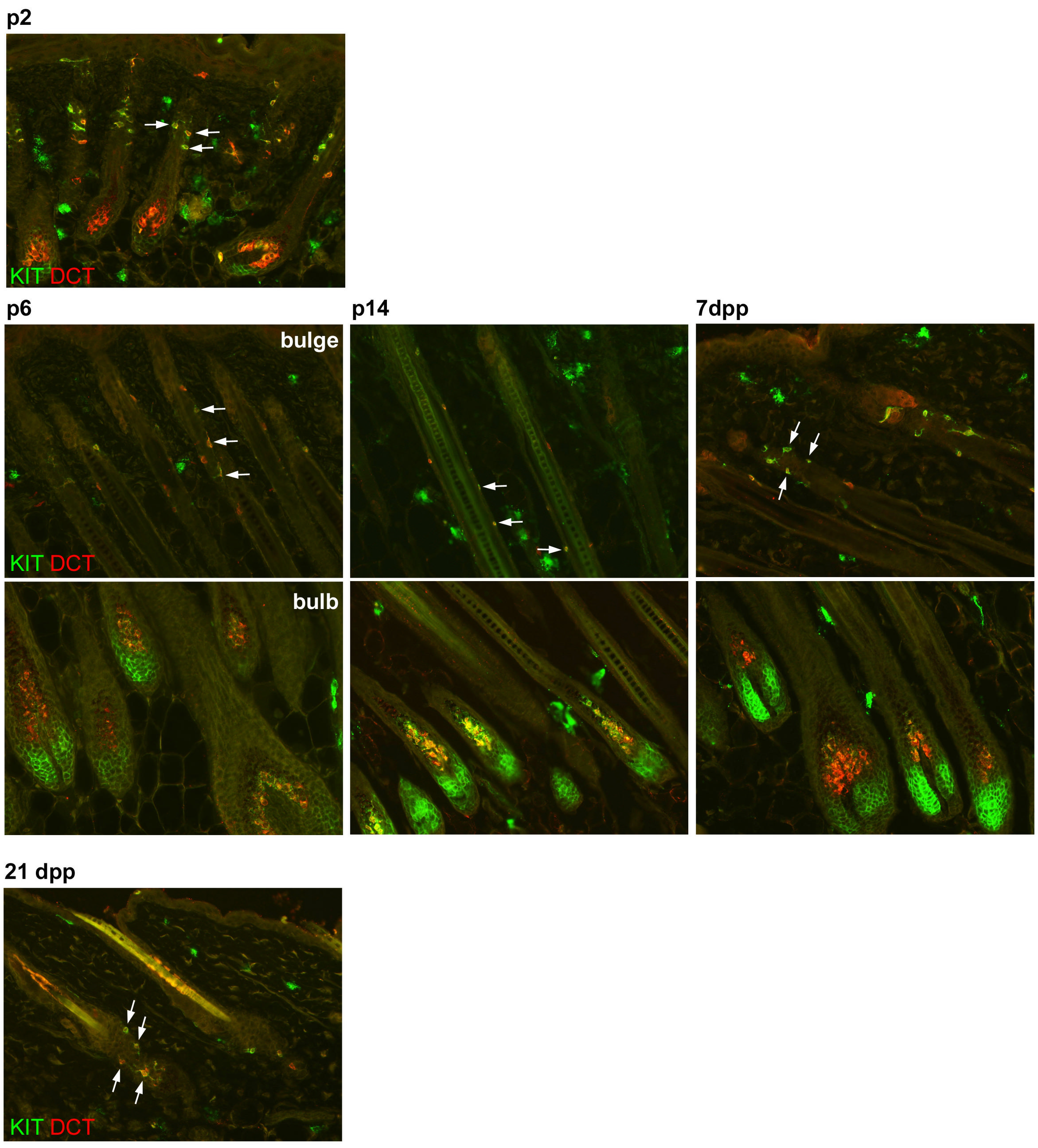

Supplement: Figure S6 — KIT expression in follicular melanocytes. Immunofluorescence staining of skins harvested at P2, P6, P14, 7dpp, and 21dpp for DCT (melanosomal, red) and KIT (membrane-bound, green). In the LPP of hairs at P2, P6, P14 and 7dpp nearly all melanocytes are KIT+, but with variable fluorescence signal intensity. At 7dpp, KIT highlights the dendricity of some LPP melanocytes. In the bulbs of P6, P14 and 7dpp hairs KIT expression is strongly localized to the keratinocytes at the bulb tip (previously reported, [71]), but is also apparent in a more diffuse, speckled pattern in the hair matrix where the differentiated melanocytes exist. KIT expression is also retained in nearly all melanocytes through catagen, shown at 21dpp. Arrows indicate examples of double-labeled cells. (TIF) [file pgen.1003644.s006.tif]

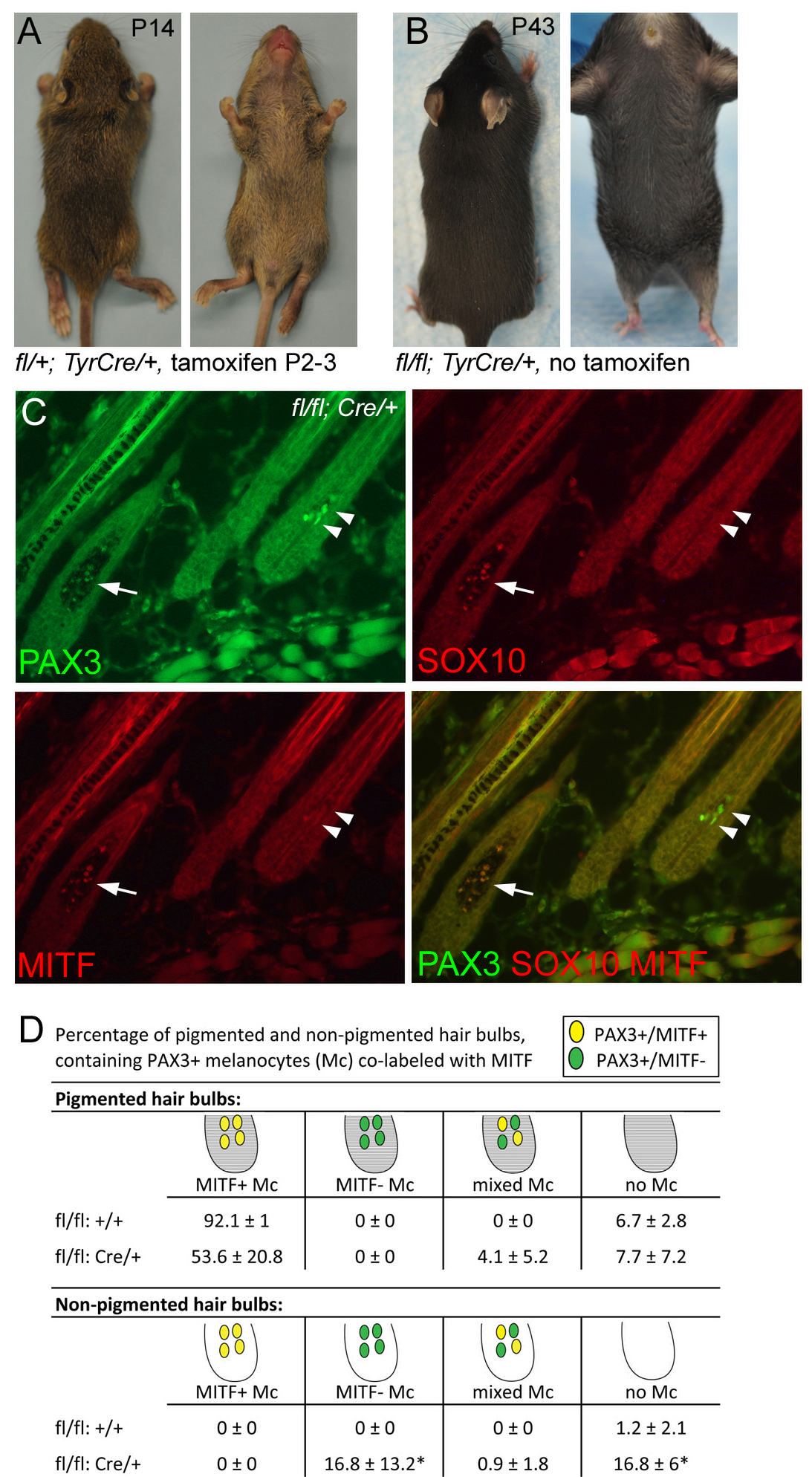

Supplement: Figure S7 — Sox10 loss results in a corresponding loss in MITF expression. (A–B) Control animals do not exhibit a hypopigmentation phenotype; (A) Sox10fl/+; Tyr::CreERT2 animal treated with topical tamoxifen at P2–3 and imaged at P14, (B) untreated Sox10fl/fl; Tyr::CreERT2 animal imaged at P43. (C) Triple-labeling of hair bulbs from Sox10fl/fl; Tyr::CreERT2 (fl/fl; Cre/+) mice described in Fig. 2E. Arrows and arrowheads indicate PAX3+/MITF+/SOX10+ and PAX3+/MITF−/SOX10− melanocytes, respectively. (D) Distribution of melanocytes double-labeled for PAX3 and MITF within pigmented (gray) and non-pigmented (white) hair bulbs in skins from Sox10fl/fl (fl/fl; +/+, n = 3) and Sox10fl/fl; Tyr::CreERT2 (fl/fl; Cre/+; n = 4) harvested on 7dpp from mice treated with TAM on 0–3dpp (*p<0.0083). (TIF) [file pgen.1003644.s007.tif]

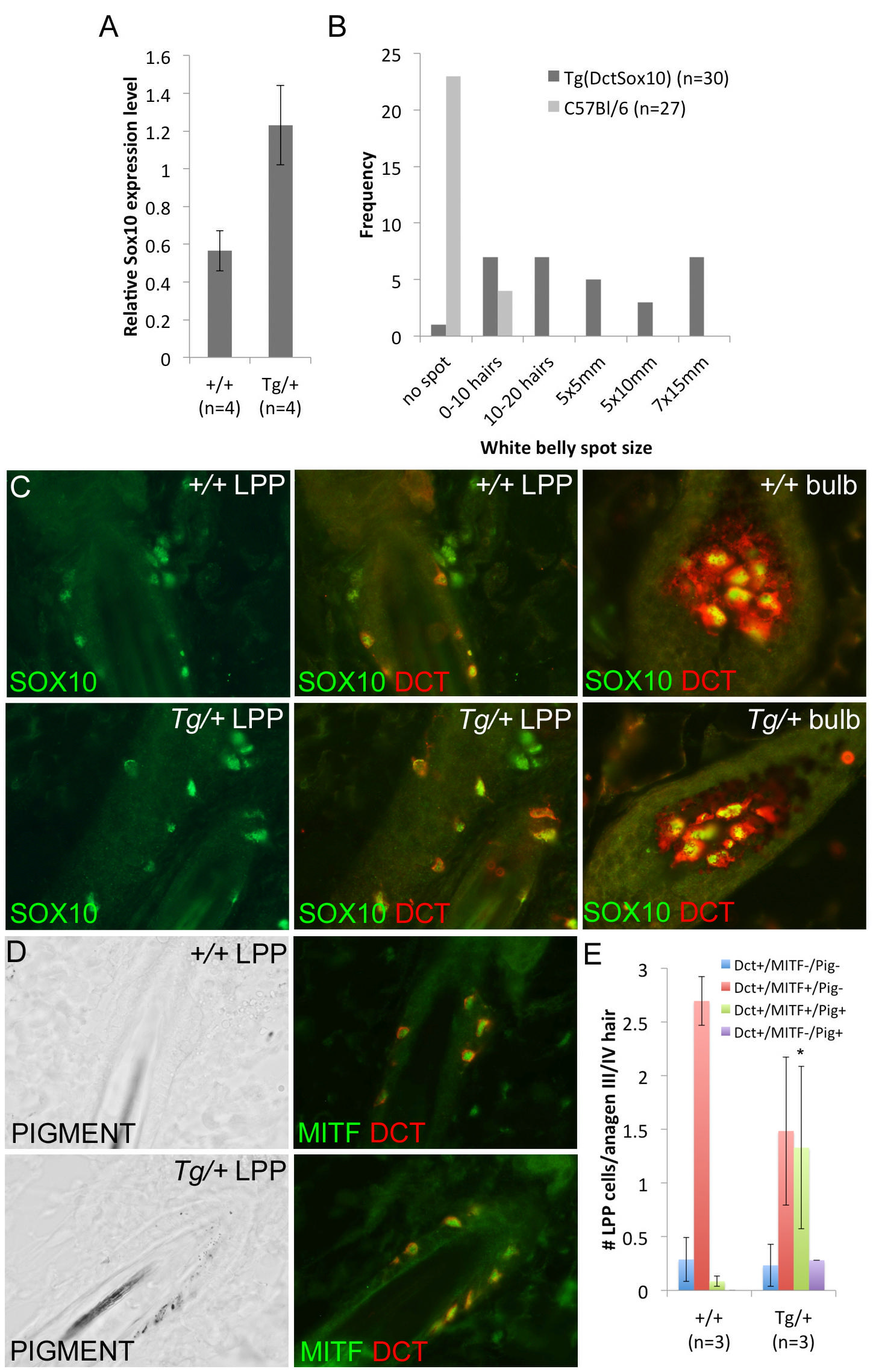

Supplement: Figure S8 — Tg(DctSox10) results in an increase in Sox10 expression, and belly spotting, but no change in the MITF expression profile of LPP melanocytes. (A) Quantitative RT-PCR of e17.5 back skin confirms that Tg(DctSox10)/+ mice exhibit a 2.4-fold increase in Sox10 mRNA levels in comparison to wild type when normalized to Pax3 (p<0.001, Student's t-test). Pax3 was used for normalization to control for possible variation in melanoblast numbers between genotypes. (B) The white belly spot observed in Tg(DctSox10)/+ mice varied in size from 0–10 white hairs up to a white spot measuring 7×15 mm. The penetrance of the white belly spot in Tg(DctSox10)/+ mice was 97% (29/30). Occasionally, a few white belly hairs were also observed in the background strain, C57Bl/6 (15%, 4/27). (C) SOX10 (green) is evident within the LPP and bulb melanocytes (DCT+, red) of both +/+ and Tg(DctSox10)/+ mice. (D) Brightfield and corresponding fluorescent images of anagen III/IV hair follicles double labeled for DCT and MITF in wild type and Tg(DctSox10)/+ animals. (E) Comparison of the number LPP melanocytes per anagen III/IV hair follicle in +/+ and Tg(DctSox10)/+ animals that express DCT, and MITF, and produce ectopic pigmentation (*p<0.0001). The average number of MITF+ melanocytes per LPP was not significantly different between Tg/+ (2.81±0.97) and wild type (2.78±0.19) animals (p = 0.96). (TIF) [file pgen.1003644.s008.tif]
