# Supplementary material for: A Dual Role for SOX10 in the Maintenance of the Postnatal Melanocyte Lineage and the Differentiation of Melanocyte Stem Cell Progenitors
Source: PLoS Genet. 2013 Jul 25;9(7):e1003644. doi: 10.1371/journal.pgen.1003644 (PMC3723529; doi:10.1371/journal.pgen.1003644)
Supplement: Table S1 — Quantitation of melanocyte immunolabeling during hair morphogenesis and hair cycling. Percentage of DCT+ melanocytes doubled labeled with the indicated marker per LPP+UTP or Bulge+SHG (∼25–50 hairs analyzed/animal, n = 3 animals per timepoint, data reported as mean ± S.D.). Grayed cells indicate the combination with the highest percentage of cells doublelabeled. *SOX10 expression in melanocytes at 21dpp is present, but weak, and normal staining protocols for DCT/SOX10 double labeling diminished visible SOX10 signal. Thus melanocytes at this timepoint were identified using KIT and then double labeled for SOX10. LPP, lower permanent portion of the hair; UTP, upper transitory portion of the hair; dpp, days post plucking; SHG, secondary hair germ of the hair. (PDF) [file pgen.1003644.s009.pdf]

## Supplemental Table 1. Quantitation of melanocyte immunolabeling during hair morphogenesis and hair cycling

| | KIT- | KITlo | KIThi | MITF- | MITF+ | TRP1- | TRP1+ | TYR- | TYR+ | SOX10- | SOX10+ |
|---|---|---|---|---|---|---|---|---|---|---|---|
| **P2 stage 4 morphogenetic hair** | | | | | | | | | | | |
| LPP | 0.23% ± 0.39% | 7.14% ± 3.89% | 92.64% ± 3.70% | 1.49% ± 1.99% | 98.51% ± 1.99% | 44.00% ± 4.00% | 56.00% ± 4.00% | 62.33% ± 3.27% | 37.67% ± 3.27% | 2.65% ± 2.29% | 97.35% ± 2.29% |
| **P2 stage 6 morphogenetic hair** | | | | | | | | | | | |
| LPP | 0.00% ± 0.00% | 14.05% ± 1.89% | 85.95% ± 1.89% | 4.47% ± 1.77% | 95.53% ± 1.77% | 64.55% ± 5.89% | 35.45% ± 5.89% | 94.79% ± 1.51% | 5.21% ± 1.51% | 3.96% ± 0.39% | 96.04% ± 0.39% |
| **p6 morphogenetic hair** | | | | | | | | | | | |
| LPP | 0.75% ± 0.78% | 28.49% ± 6.79% | 33.93% ± 6.26% | 1.86% ± 1.94% | 56.33% ± 7.35% | 50.51% ± 5.66% | 4.01% ± 1.39% | 56.46% ± 9.44% | 1.14% ± 1.38% | 0.79% ± 0.71% | 65.05% ± 2.70% |
| UTP | 2.52% ± 2.01% | 22.49% ± 2.35% | 11.81% ± 2.24% | 0.51% ± 0.44% | 41.30% ± 5.62% | 36.89% ± 4.57% | 8.58% ± 2.69% | 42.40% ± 8.07% | 0.00% ± 0.00% | 0.46% ± 0.80% | 33.70% ± 1.50% |
| **p14 morphogenetic hair** | | | | | | | | | | | |
| LPP | 3.54% ± 2.70% | 36.72% ± 6.11% | 15.48% ± 2.31% | 0.47% ± 0.67% | 57.88% ± 4.86% | 45.38% ± 3.59% | 12.58% ± 2.70% | 49.97% ± 6.74% | 1.21% ± 0.22% | 4.16% ± 4.22% | 49.53% ± 8.59% |
| UTP | 5.67% ± 1.02% | 32.58% ± 1.70% | 6.00% ± 1.41% | 0.00% ± 0.00% | 41.65% ± 5.53% | 13.20% ± 2.87% | 28.84% ± 2.53% | 46.40% ± 6.07% | 2.42% ± 0.45% | 2.14% ± 1.65% | 44.17% ± 4.61% |
| **7dpp (~adult anagen IV/V)** | | | | | | | | | | | |
| LPP | 0.00% ± 0.00% | 10.57% ± 5.31% | 64.55% ± 2.69% | 10.30% ± 5.45% | 54.07% ± 5.48% | 38.27% ± 4.25% | 43.27% ± 7.22% | 49.97% ± 6.74% | 1.21% ± 0.22% | 10.99% ± 7.84% | 65.59% ± 3.59% |
| UTP | 0.00% ± 0.00% | 4.30% ± 3.50% | 20.57% ± 4.01% | 4.52% ± 0.99% | 31.11% ± 0.96% | 4.08% ± 1.49% | 14.38% ± 6.84% | 46.40% ± 6.07% | 2.42% ± 0.45% | 2.40% ± 0.10% | 21.02% ± 9.84% |
| **21dpp (~adult catagen VII)** | | | | | | | | | | *compared to KIT | |
| Bulge | 0.74% ± 0.69% | 16.68% ± 2.44% | 36.92% ± 3.27% | 49.41% ± 9.52% | 4.77% ± 1.85% | 25.74% ± 6.02% | 14.21% ± 3.03% | 39.51% ± 9.85% | 0.00% ± 0.00% | 1.78% ± 2.08% | 56.34% ± 17.08% |
| SHG | 2.26% ± 1.96% | 11.48% ± 4.67% | 31.91% ± 11.91% | 34.72% ± 8.20% | 11.10% ± 5.42% | 16.39% ± 5.54% | 43.65% ± 11.88% | 58.73% ± 8.20% | 1.75% ± 2.42% | 2.17% ± 0.78% | 39.71% ± 15.56% |
